# Sustainable Synthesis of Terpolyesters Based on a Levoglucosenone-Derived Cyclic Acetal Diol

**DOI:** 10.1021/acssuschemeng.4c10010

**Published:** 2025-03-04

**Authors:** Giacomo Lombardo, Cicely M. Warne, Giacomo Damonte, Anamaria Todea, Lajos Nagy, Georg M. Guebitz, Florent Allais, Sami Fadlallah, Alessandro Pellis

**Affiliations:** † University of Genova, Department of Chemistry and Industrial Chemistry, via Dodecaneso 31, Genova (GE) 16146, Italy; ‡ BOKU University, Vienna, Department of Agrobiotechnology, IFA-Tulln, Institute of Environmental Biotechnology, Konrad-Lorenz-Strasse 20, Tulln an der Donau 3430, Austria; § ACIB GmbH, Konrad-Lorenz-Strasse 20, Tulln an der Donau 3430, Austria; ∥ University Politehnica Timisoara, Faculty of Chemical Engineering, Biotechnology and Environmental Protection, Vasile Parvan 6, Timisoara 300223, Romania; ⊥ University of Debrecen, Department of Applied Chemistry, Egyetem tér 1, Debrecen H-4032, Hungary; # URD Agro-Biotechnologies Industrielles (ABI), CEBB, AgroParisTech, Pomacle 51110, France

**Keywords:** levoglucosenone-derived diols, biocatalytic synthesis, terpolymers, enzymatic copolymerization, cellulose-derived green solvents

## Abstract

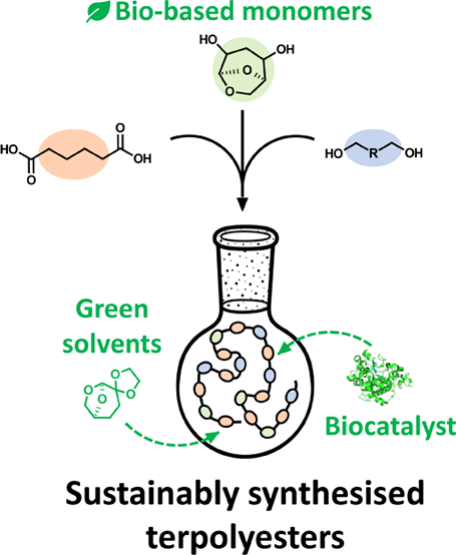

Biobased polyesters are gaining increasing interest as sustainable replacements for traditional fossil-based polymers. The compound (1R,2S,5R)-6,8-dioxabicyclo[3.2.1]octane-2,4-diol (HO-LGOL) is a cellulose-derived monomer that can be used to synthesize polyesters with properties similar to those obtained with classical reagents. In this work, several HO-LGOL-based copolymers were sustainably synthesized in the green solvent dioxolane Cygnet (0.0) (referred to as “Cygnet 2”) utilizing *Candida antarctica* Lipase B (CaLB) as a biocatalyst. HO-LGOL was reacted with dimethyl adipate and aliphatic diols of various lengths. Different ratios of reactants were also investigated, and an equimolar amount of HO-LGOL and aliphatic diol was found to yield copolymers with the highest level of HO-LGOL incorporation. Matrix-assisted laser desorption ionization-time-of-flight mass spectrometry (MALDI-TOF) confirmed the structure of end groups and the presence of HO-LGOL in longer polymer chains. The incorporation of HO-LGOL resulted in terpolymers with an HO-LGOL content of up to 49% (relative to the aliphatic diol), which exhibited lower crystallinity and higher thermal stability compared to the corresponding aliphatic homopolymers.

## Abstract 2

This work investigates the sustainable enzyme-catalyzed synthesis of terpolyesters, utilizing both cellulose-derived monomers and cellulose-derived green solvents.

## Introduction

In recent years, due to the increasingly evident environmental impact of toxic compounds and materials, and the consequent restrictions imposed by EU legislation through Registration, Evaluation, Authorization, and Restriction of Chemicals (REACH), research efforts in developing novel sustainable materials have increased exponentially. One such area of research is the development of sustainable polymer materials. Around 99% of polymers are either derived from finite fossil fuels, are nonbiodegradable, or both,^1^ making these materials unsustainable at every point of their lifecycle. In addition to developing new strategies for dealing with end-of-life issues, new renewable feedstocks must be investigated to make the synthesis of polymer materials more environmentally friendly.

**Figure 1:**
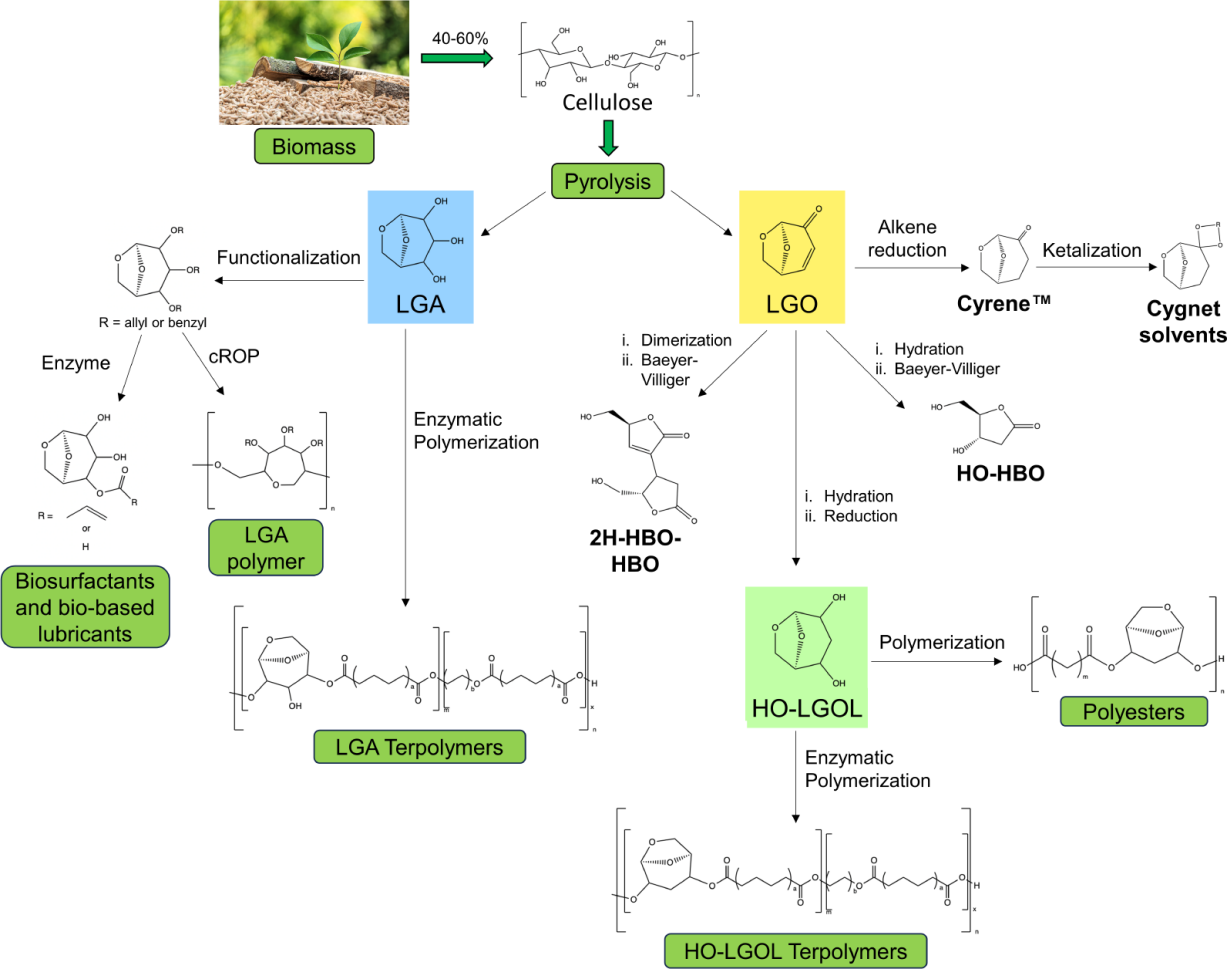
Synthetic pathways to notable compounds and polymers derived from LGA and LGO, showing their structures and reaction steps.^2,3,11−20^

The development of materials derived from biomass, and therefore referred to as “biobased”, constitutes a major part of research in sustainable chemistry. Given that cellulose is the most abundant biopolymer in nature, many biobased chemical platforms focus on its valorization. Two such chemicals that can be produced from cellulose are levoglucosan (LGA) and levoglucosenone (LGO)^2,3^ (highlighted in blue and yellow, respectively, in Figure 1), both of which exhibit chemical structures and functionalities useful for synthesizing biobased polymers. The yield of LGA from cellulose is generally higher than LGO,^3^ but LGA can be dehydrated to give LGO in the presence of an acid catalyst.^4^ LGO is a highly desired platform chemical, as it can be used to synthesize many other materials, such as chiral auxiliaries and ligands,^5^ organocatalysts,^6,7^ potential drug molecules^8^ and natural product-like compounds.^9,10^

LGO can be obtained from cellulose via the Furacell process.^2^ It is a precursor to the green solvent Cyrene (Figure 1), a dipolar aprotic solvent with the potential to replace toxic solvents like dimethylformamide (DMF) and *N*-methyl-2-pyrrolidone (NMP).^15^ Cyrene can be further functionalized through the reaction of the ketone group with a diol to generate a ketal, resulting in a class of solvents called the Cygnets. Based on ACD/l-lab predictions, these solvents should be nongenotoxic and nonmutagenic, and have previously been investigated for membrane casting and enzymatic polycondensation applications.^16,17^ LGO can also be chemically derivatized to form different monomers suitable for polymerization. 2-Deoxy-d-ribonolactone (HO-HBO), shown in Figure 1, can be synthesized from LGO in a two-step reaction and has been polymerized with diacyl chlorides to form polyesters.^18^ Similarly, the monomer (S)-5-(hydroxymethyl)-3-[(2S,3S)-2-(hydroxymethyl)-5-oxotetrahydrofuran-3-yl]furan-2(5H)-one (2H-HBO-HBO) (Figure 1) can also be synthesized from LGO and has been used to synthesize polyesters, both enzymatically^17^ and chemically.^21^

Another interesting monomer obtained from LGO via a one-pot, two-step process of hydration-reduction is (1R,2S,5R)-6,8-dioxabicyclo[3.2.1]octane-2,4-diol (HO-LGOL), whose structure can be seen in Figure 1 (highlighted in light green). HO-LGOL is a diol containing only secondary hydroxyl groups and has been used to synthesize polyesters via reaction with diacyl chlorides or diesters (in the presence of a metal catalyst). Interestingly, Diot-Néant et al. succeeded in producing materials with high thermal stabilities, even at lower molecular weights.^12^ The thermal stability of these HO-LGOL-based polymers makes them interesting candidates for further applications, but almost all polymers were poorly soluble in common solvents,^12^ making them difficult to analyze and process.

LGA (Figure 1, highlighted in blue) is analogous to HO-LGOL; characterized by three instead of two hydroxyl groups on the pyranose ring, all of which are capable of hydrogen bonding, limiting its reactivity and lengthening reaction times. Similar to HO-LGOL, the rigid bicyclic structure is interesting for polymer chemistry, as it can produce materials with relatively high *T*_g_.^3,11^ By alkylating the hydroxyl moieties of LGA with allylic or benzyl functional groups, it is possible to synthesize polymers with a thermal stability above 300 °C through cationic ring-opening polymerization.^14^ LGA can also be functionalized through esterification of the hydroxyl groups to create biosurfactants and biobased lubricants.^13,22^ One particularly notable example of LGA-based polymers can be found in the work of Bassut et al., who synthesized terpolymers by copolymerizing LGA with diethyl sebacate or adipate, and aliphatic diols of different lengths (4–12 carbons).^23^ The polyesters prepared in this work achieved a number-average molecular weight (*M*_n_) up to 7900 Da, and they were able to incorporate LGA into the polymer chain up to 35% (with respect to the aliphatic diol). Interestingly, this was done through the use of a biocatalyst, namely *Candida antarctica* Lipase B (CaLB), despite its predilection for primary hydroxyl groups,^23−26^ suggesting that this may also be a viable procedure to apply to the HO-LGOL monomer.

CaLB is a widely used lipase due to its broad substrate scope, thermal stability, and remarkable catalytic activity. There have been many efforts to find enzymes that can compete with or even exceed CaLB in terms of catalytic activity and enantioselectivity in transesterification reactions. Novel enzymes have been screened for their ability to synthesize polyesters,^27^ and techniques such as enzyme engineering can alter the substrate selectivity of the enzyme.^28^ Despite this, the ability of CaLB to synthesize high molecular weight polyesters (Mn > 40 kDa),^29,30^ as well as its commercial availability, has ensured it remains the enzyme of choice for many researchers.

**Figure 2:**
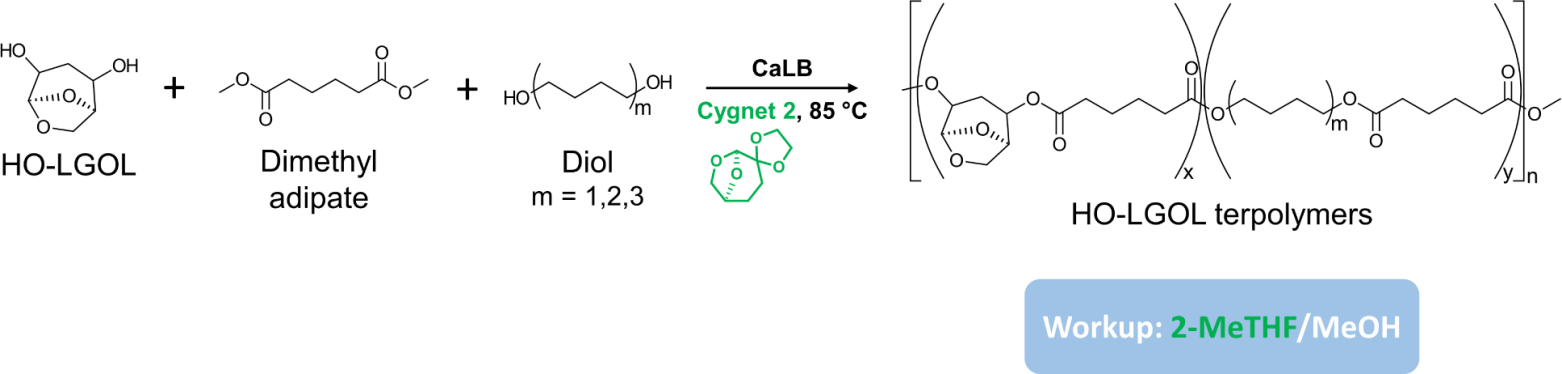
Scheme of the synthetic procedure developed in this work, showing the reaction of the levoglucosenone-derived cyclic acetal diol, HO-LGOL, dimethyl adipate (DMA) and three different aliphatic diols in the presence of CaLB and Cygnet 2 to form terpolyesters, worked up in 2-MeTHF and methanol (MeOH).

Taking into account the previously described work on LGA,^23^ and with the aim of introducing a high content of HO-LGOL into a polymer formulation, in this study we developed novel terpolyesters based on dimethyl adipate, HO-LGOL, and various linear aliphatic diols (C4–C12) (Figure 2). In addition, the synthesis was made environmentally friendly, first through the use of enzymes, and second through the replacement of diphenyl ether (DPE), a commonly used solvent in enzymatic polycondensation reactions,^31^ and chloroform, which is used in the workup procedure, with dioxolane Cygnet (0.0) (referred to as Cygnet 2) and 2-methyl-tetrahydrofuran (2Me-THF), respectively. Indeed, Cygnet 2 is a biobased solvent that has been successfully used in enzymatic polycondensations, outperforming DPE in terms of the molecular weight of the synthesized polymers,^17^ while 2Me-THF is much less hazardous^32^ compared to chloroform. In detail, the effects of aliphatic diol length and the ratio between monomers were investigated by characterizing the terpolymers in terms of the percentage of incorporation of HO-LGOL into the polymer chain, as well as yield and molecular weight.

## Materials and Methods

### Chemicals and Materials

1,4-Butanediol (99%, ReagentPlus), 1,8-octanediol (98%), 1,12-dodecanediol (99%), *Candida antarctica* lipase B immobilized on acrylic resin beads (≥5000 U/g, Code L4777), and methanol (≥99.9%) were purchased from Sigma-Aldrich. Dimethyl adipate (99%) and 2-methyltetrahydrofuran (99%) were purchased from Alfa Aesar (now rebranded as Thermo Scientific). Cygnet 2^17^ and HO-LGOL^12^ were synthesized according to previously published procedures.

### Optimized Terpolyester Synthesis

In a 25-mL round-bottom flask, dimethyl adipate (522.6 mg, 3 × 10^–3^ mol) and HO-LGOL were withdrawn with CaLB (10% by weight of monomers) and Cygnet 2 (1.7 g) and heated to 85 °C at 400 rpm (12 mm stir bar) for 4 h. The aliphatic diol was then added (either 1,4-butanediol, 1,8-octanediol, or 1,12-dodecanediol), and the reaction was left for a further 4 h. After this phase, the system was placed under a dynamic vacuum (20 mbar) for a further 88 h (total reaction time 96 h). For the workup procedure, 2 mL of 2-MeTHF was added to the reaction medium to solubilize the formed polymer, and the immobilized enzyme was removed by filtration through cotton packed into a 150-mm glass Pasteur pipette. The enzyme was further washed with 3 × 1 mL 2-MeTHF to remove all polymer. Subsequently, the polymer solution was added to 35 mL ice-cold MeOH to induce precipitation. Samples were then vortexed and centrifuged (3700 rpm, 4 °C, 10 min). The supernatant was discarded, and the centrifugation step was repeated two times, each with 20 mL ice-cold MeOH. Polymers were dried under vacuum before being fully characterized.

### Nuclear Magnetic Resonance (NMR) Spectroscopy

NMR spectra were acquired either using a JEOL 400 MHz spectrometer at room temperature using the deuterated solvent CDCl_3_ with tetramethylsilane (TMS, 0.03%) as a reference, with ^1^H spectra that were acquired at 400 MHz and ^13^C spectra at 75 MHz, or on a Bruker Avance II 400 at room temperature with standard Bruker pulse programs. The samples were prepared by dissolving ∼10 mg of the polymer in 0.6 mL of CDCl_3_. Spectra were reported with the chemical shift *(*δ) in ppm (normalized on the signal of TMS (0.00 ppm)) on the *x*-axis and the signal intensity on the *y*-axis.

### NMR Characterization

#### Poly(HO-LGOL adipate-*co*-1,4-octylene adipate)

^1^H NMR: (400 MHz, CDCl_3_, 27 °C) δ 5.44 (s, 1H), 4.86 (s, 1H), 4.60 (s, 1H), 4.09 (s, 4H), 3.87 (m, 2H), 3.68 (m, 1H), 2.35 (m, 8H), 2.09 (m, 1H), 1.94 (m, 1H), and 1.67 (m, 12H).

^13^C NMR: (101 MHz, CDCl_3_, ppm*)* δ 173.46, 100.31, 77.43, 77.12, 76.80, 74.46, 69.62, 68.92, 66.69, 63.99, 33.94, 27.48, 25.37, 25.17, 24.45, 24.35, and 24.10.

#### Poly(HO-LGOL adipate-*co*-1,8-octylene adipate)

^1^H NMR: (400 MHz, CDCl_3_, 27 °C, ppm) δ 5.44 (s, 1H), 4.86 (s, 1H), 4.60 (s, 1H), 4.09 (s, 4H), 3.87 (m, 2H), 3.68 (m, 1H), 2.35 (m, 8H), 2.09 (m, 1H), 1.94 (m, 1H), 1.67 (m, 8H), and 1.33 (s, 8H).

^13^C NMR: (101 MHz, CDCl_3_, ppm*)* δ 173.54, 100.32, 77.43, 77.11, 76.79, 74.46, 69.61, 68.91, 66.68, 64.56, 34.02, 29.20, 28.67, 27.49, 25.92, and 24.50.

#### Poly(HO-LGOL adipate-*co*-1,12-dodecylene adipate)

^1^H NMR: (400 MHz, CDCl_3_, 27 °C, ppm) δ 5.44 (s, 1H), 4.86 (s, 1H), 4.60 (s, 1H), 4.09 (s, 4H), 3.87 (m, 2H), 3.68 (m, 1H), 2.35 (m, 8H), 2.09 (m, 1H), 1.94 (m, 1H), 1.67 (m, 8H), and 1.33 (s, 16H).

^13^C NMR: (101 MHz, CDCl_3_, ppm*)* δ 173.58, 100.32, 77.45, 77.13, 76.81, 74.45, 69.59, 68.89, 66.68, 64.65, 51.65, 34.03, 29.64, 29.60, 29.35, 28.70, 27.48, 25.99, and 24.51.

### Gel Permeation Chromatography (GPC)

Polymers were dissolved in CHCl_3_ to a concentration of ∼2 mg/mL and filtered through cotton wool packed into a 150-mm glass Pasteur pipette. The analysis was performed at 30 °C on an Agilent Technologies 1260 Infinity HPLC System equipped with a 17.369 6.0 mm ID × 40 mm LHHR-H, 5 μm guard column and an 18,055 7.8 mm ID × 300 mm L GMHHR-N, 5 μm TSK gel liquid Tosoh Bioscience chromatography column, using CHCl_3_ as the eluent at a flow rate of 1 mL/min for a total time of 20 min. An Agilent Technologies G1362A refractive index was used as the detector. The calibration curve was obtained using polystyrene standards in the 250–70 ,000 Da molecular weight range.

### Differential Scanning Calorimetry (DSC)

DSC analyses were performed on a DSC 1 Mettler Toledo. A polymer sample of ∼5 mg was heated from 25 to 150 °C, held at 150 °C isothermally for 2 min, then cooled to −100 °C and heated again to 150 °C, always using a heating/cooling rate of 10 °C/min. Measurements were performed in an inert atmosphere under a constant N_2_ flow of 20 mL/min.

### Thermogravimetric Analysis (TGA)

TGA was performed on a TGA/DSC 1 Mettler Toledo. ∼10 mg of sample was added to a 40 μL aluminum crucible, placed into the furnace with an inert gas flow (N_2_) of 80 mL/min and heated from 25 to 800 °C using a heating rate of 10 °C/min.

### Matrix-Assisted Laser Desorption Ionization (MALDI)

Analysis was carried out using a Bruker autoflex speed MALDI-TOF/TOF mass spectrometer equipped with a time-of-flight (TOF) mass analyzer. 2,5-Dihydroxybenzoic acid (DHB) was used as the matrix (20 mg/mL in THF), and sodium trifluoroacetate (NaTFA) as the ionization agent (5 mg/mL in THF). Samples were diluted to 1 mg/mL in THF and mixed with DHB and NaTFA in a 50/10/5 (DHB/sample/NaTFA) ratio by volume. For the analysis, 0.5/1.0 μL of this mixture was dropped onto the sample carrier, and the measurement was conducted with the detector set in both linear and reflectron modes.

## Results and Discussion

### Optimization of Poly(HO-LGOL Adipate-co-1,8-octylene Adipate) Synthesis

Polyesters were first synthesized using a one-step, one-pot procedure in which all monomers were mixed simultaneously in a reaction vessel, using 1,8-octanediol (ODO) and HO-LGOL as the aliphatic diols and dimethyl adipate (DMA) as the diester. The molar ratio between the monomers was arbitrarily set at 100:25:50 (DMA:HO-LGOL:ODO), with a small excess of DMA.

**Figure 3:**
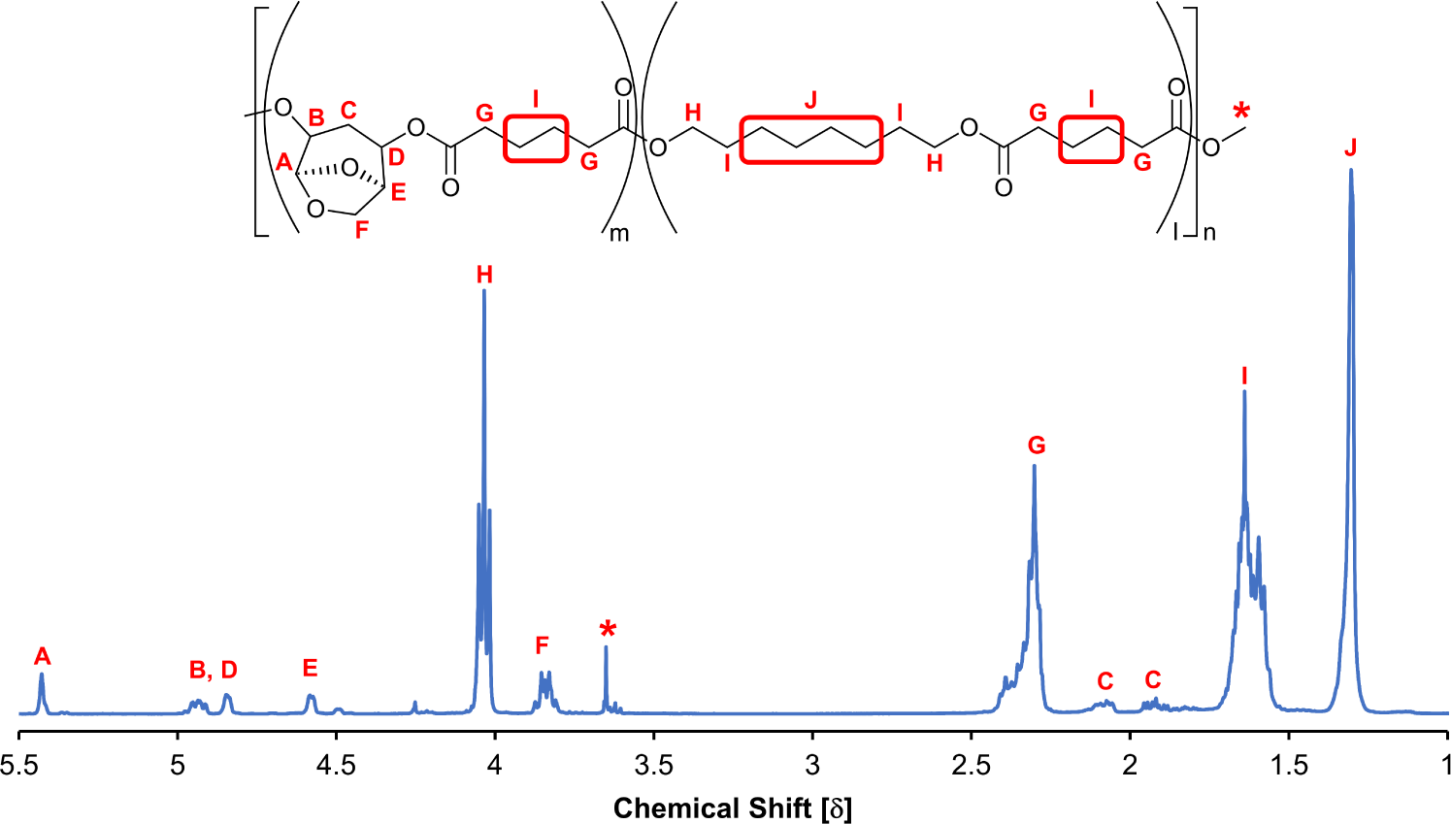
Fully assigned ^1^H NMR spectra of poly(HO-LGOL adipate-*co*-1,8-octylene adipate) synthesized using 100:25:50 (DMA:HO-LGOL:ODO) ratio in one-pot procedure. Asterisk denotes peaks from the −CH_2_OH and −OCH_3_ end groups protons. ^13^C and HSQC spectra can be found in Supporting Information (Figures S1 and S9).

The percentage of HO-LGOL incorporated into the polymer was calculated using eq 1, with reference to Figure 3. This value indicates the incorporation of HO-LGOL relative to the aliphatic diol rather than the overall percentage of HO-LGOL in the polymer. The results of this first synthesis gave a low yield of 14%, and the percentage of incorporated HO-LGOL was determined to be 26% (Figures 3 and S1).
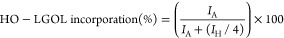
1

Considering the low yield and the limited incorporation of HO-LGOL into the polymer chain obtained with this approach, the procedure was modified to optimize these factors. The new procedure maintained the same molar ratio as the previous experiment, but reacted DMA with HO-LGOL for the first 4 h, before adding the aliphatic diol (ODO). The reaction was then left to run for another 4 h, upon which reduced pressure was applied. This modification ensured that HO-LGOL did not have to compete with the more easily polymerized ODO during the first 4 h of the polycondensation. Various ratios of HO-LGOL with respect to the ratios of the corresponding compounds of ODO and DMA were also investigated. The amount of diester was kept constant, while the amounts of HO-LGOL and ODO were varied, as shown in Table 1.

**Table 1:** HO-LGOL Incorporation, Yield, Molecular Weight, Degree of Polymerization (DP) and Dispersity (*Đ*) Data Obtained for Poly(HO-LGOL Adipate-*co*-1,8-octylene Adipate) Synthesized in Cygnet 2 as the Solvent and with CaLB as the Biocatalyst at 85 °C

Entry	Monomer ratio [DMA:HO-LGOL:ODO]	HO-LGOL content [%]^a^^a^	Yield [%]^b^^b^	*M*_n_ [Da]^c^^c^	*M*_w_ [Da]^c^^c^	Đ^c^	DP^d^^d^
1	100:25:50	24	32	3000	9700	3.20	11.9
2	100:50:75	12	52	2400	5700	2.41	9.2
3	100:50:50	42	41	1200	4400	3.80	4.5

a Determined via ^1^H NMR analysis (Figures S2–S4).

b Determined by gravimetric measurements.

c Determined via gel permeation chromatography analysis.

d Calculated using eq S1 and Table S1.

Notably, when comparing the new procedure with the previous one, although the yield increased considerably, HO-LGOL incorporation remained almost the same (a negligible decrease from 26% to 24% was observed). Bassut et al. suggested that LGA reacts more readily with oligomers compared to monomers,^23^ and a previous work that compared the enzymatic polycondensation of a bulky rigid monomer with a flexible aliphatic one observed that higher molecular weights were obtained for the bulky monomer.^33^ Computational analysis suggested that dimers containing the bulkier monomer had more conformations compatible with the elongation reaction when in the active site.^33^

Regarding the ratio of reactants, it can be seen from Table 1 that the optimum ratio differs depending on the parameter considered. The use of an equimolar ratio (entry 3) led to 42% HO-LGOL incorporation, the highest for this set of reactions. Conversely, an excess of ODO (entry 2) resulted in the highest yield, and an excess of diester (entry 1) gave higher molecular weights (9700 Da weight-average molecular weight). As the main goal of this polymerization is the synthesis of HO-LGOL-based polyesters, the yield and molecular weights of these polymers should be considered with respect to HO-LGOL incorporation, as high yields or molecular weights are insignificant if chains consist primarily of the recombination of the ODO–DMA repeat units. Although the polymer in entry 2 has the highest yield, it is also characterized by the lowest percentage of HO-LGOL. The excess of aliphatic ODO, a substrate preferred by CaLB,^34^ results in the incorporation of this monomer into the polymer instead of HO-LGOL. The results of entry 3, with a lower yield but higher incorporation of HO-LGOL, are more preferable. Looking at the molecular weight, the 25:50 ratio (entry 1) resulted in the highest values, with a dispersity of 3.20. The other two ratios exhibit lower values, with the 50:75 ratio having the second-highest *M*_n_ and *M*_w_ values for this set of experiments. However, due to the low HO-LGOL incorporation, this polymer mostly consists of ODO-DMA units.

### MALDI Analysis of ODO-Based Terpolymers

MALDI analysis of the synthesized ODO-based terpolymers was performed, providing further insights into the polymer structure, particularly end group analysis. All three polyesters were analyzed in both linear and reflectron modes. The three different units (HO-LGOL, DMA, and ODO) have the same molecular weight (128 Da); therefore, the weight of both possible diol+diester units (ODO+DMA or HO-LGOL+DMA) is also the same, at 256 Da. Several combinations of end groups were detected (series A–E), as reported in Table 2. Methyl ester end groups were readily hydrolyzed to carboxylic acids in many cases.

**Table 2:**
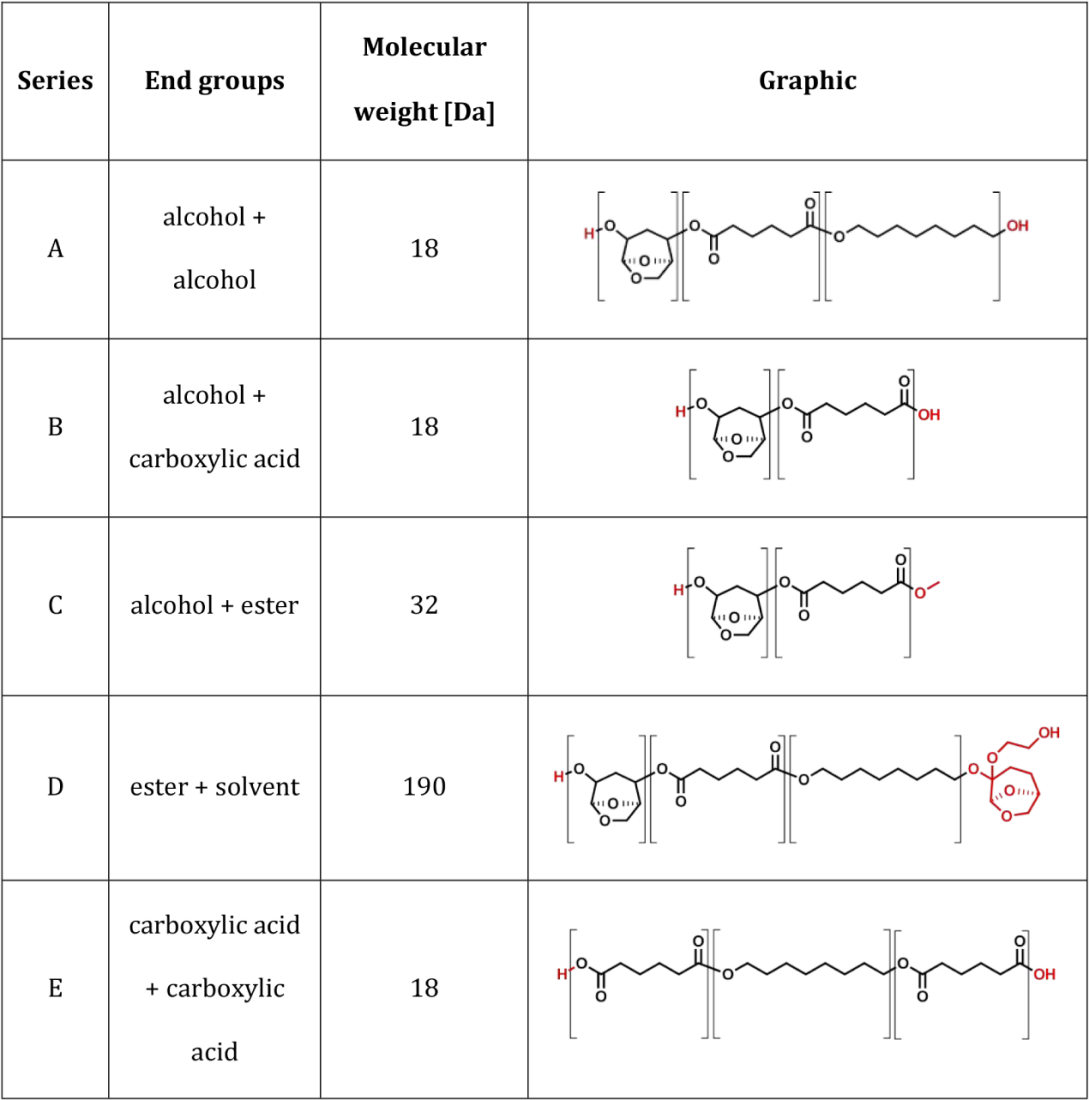
End Groups Observed in MALDI Analysis of Enzymatically Synthesized Poly(HO-LGOL Adipate-*co*-1,8-octylene Adipate) at Different Molar Ratios, Labeled A–E, Indicating the Group on Each End, the Combined Molecular Weight of Those Groups, and a Graphical Representation of Possible Polymer Repeat Units, with End Groups Highlighted in Red

The reflectron analysis of entry 2 expects a difference between the neighboring peaks to be the expected 256 Da. Among all of the peaks, those of series A possess the highest intensity, which is expected for a polyester synthesized with an excess diol. Several peaks belonging to series B were also detected, corresponding to both [R-COOH + Na]^+^ and [R-COONa + Na]^+^ ions, with a difference of 22 Da between them. Not all diester end groups underwent hydrolysis; series C indicates the presence of polymer chains with both a diol and diester end group, although in smaller quantities. Series D possibly indicates products of a side reaction between the polymer and the Cygnet 2 solvent, with the probable end group structure seen in Table 2. The ketal ring is known to be labile in acidic environments, and ring-opening is not expected under normal conditions; however, this series was only detected at very low intensity.

**Figure 4:**
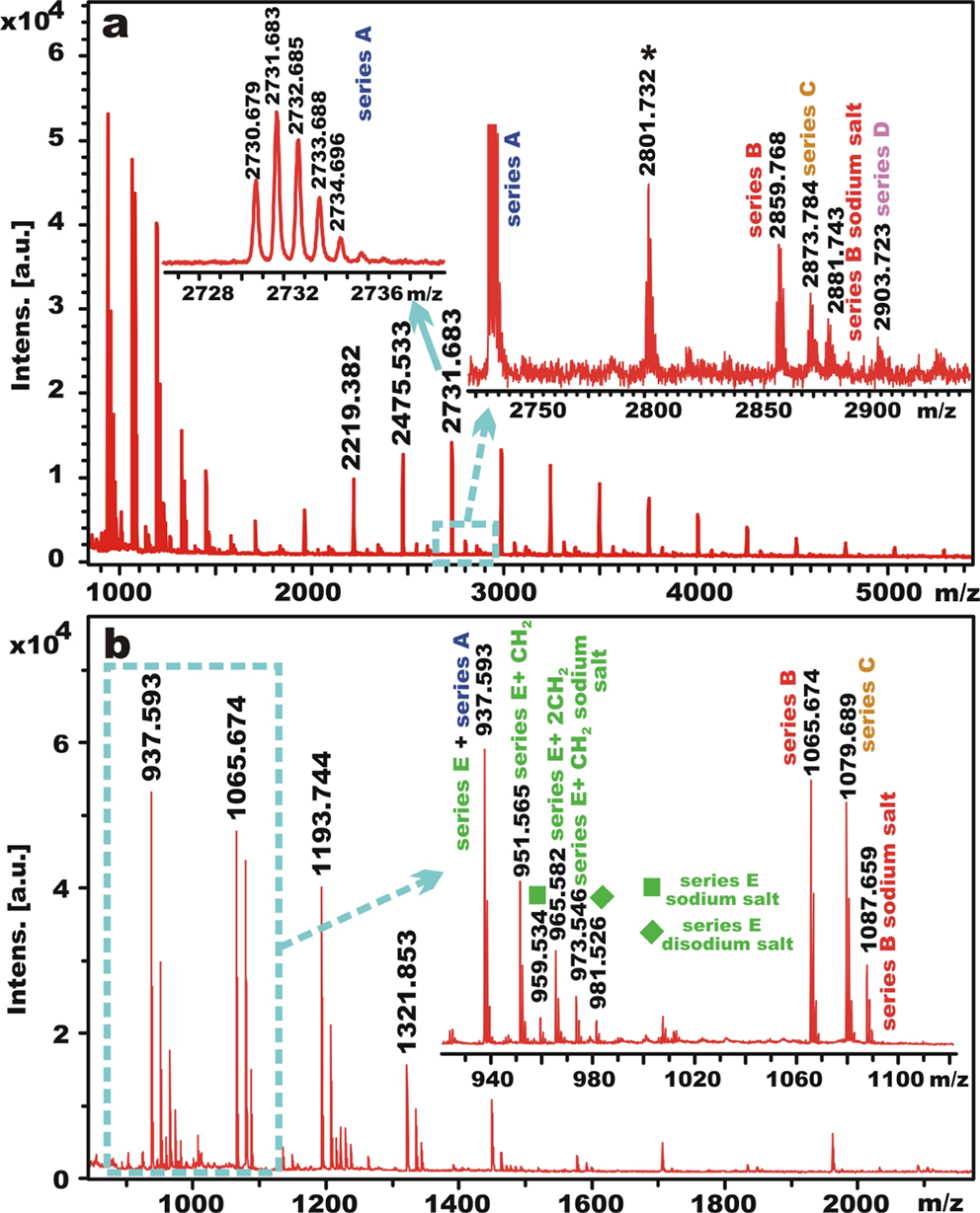
MALDI spectra of poly(HO-LGOL adipate-*co*-1,8-octylene adipate) synthesized using a 100:50:75 monomer ratio [DMA:HO-LGOL:ODO] (entry 2, Table 1) in reflectron mode showing (a.) *m*/*z* 900–5400, and (b) a close up of *m*/*z* 900–2100.

Additional series were observed under 1500 *m*/*z*, as shown in Figure 4. The end groups of series E have the same nominal molecular weights as those of series A, but they can be distinguished by the presence of additional peaks at +14 and +28 Da (attributed to methylation of one or both end groups). Taking the peak at *m*/*z* 937 as an example (Figure 4b), when one end group is methylated, the mass increases by 14 Da (*m*/*z* 951), and when both ends are methyl esters, the *m*/*z* value is 965.

Comparing these results with entries 1 and 3 (Table 1), we can observe the same series at different intensities. For entry 1 (Table 1), most of the peaks observed in the low *m*/*z* region belong to the E and B series. In higher *m*/*z* region, series E and its methyl ester forms (+14 Da and +28 Da) were detected. This is to be expected for a polymer synthesized using an excess of DMA. For entry 3 (Table 1), the polyester synthesized using equimolar amounts of diester and diol showed that series E and C were the most significant (Figure S11), and in linear mode, both series were detected up to 8000 Da (Figure S12). Interestingly, the mass resolution of the mass spectrometer at lower molecular weights was sufficient to observe the shoulders on the peaks caused by the different ratio of diols (HO-LGOL/ODO) in oligomer chains. By taking the peak at *m*/*z* 2475.303 (Figure S13) as representative of series A (assuming no overlap with series E), it is possible to calculate the HO-LGOL and ODO values, which are theoretically closest to the measured mass. In particular, this chain contains 9 DMA units, 4–5 HO-LGOL, and 5–6 ODO, indicating 40–50% HO-LGOL incorporation. Alternatively, if *m*/*z* 2475 is regarded as series E (assuming no overlap with series A), it contains 10 DMA, 3–4 HO-LGOL, and 5–6 ODO, giving an HO-LGOL incorporation of 33–44%. By comparison of these results, it is interesting to note that a significant amount of HO-LGOL is present in the longer chains rather than just the shorter oligomers, as the average degree of polymerization (DP) of this polyester was 4.5 (Table 1).

### Synthesis of Terpolyesters with Aliphatic Diols of Different Length

As seen from the previous sections, when considering only the incorporation of HO-LGOL, the equimolar ratio (entry 3, Table 1) was the optimum, followed by the ratio with excess DMA (entry 1, Table 1). These two ratios (25:50 and 50:50 of HO-LGOL:aliphatic diol) were further investigated in the polycondensations with diols of different chain lengths, namely, 1,4-butanediol (BDO) and 1,12-dodecanediol (DOD). The results of these experiments are reported in Table 3 and Figure S16.

**Table 3:** HO-LGOL Incorporation, Yield, Molecular Weights, Degree of Polymerization (DP) and Dispersity for Poly(HO-LGOL Adipate-*co*-1,4-butylene adipate) and Poly(HO-LGOL Adipate-*co*-1,12-dodecylene Adipate) that were Enzymatically Synthesized using Different Monomer Ratios

Aliphatic diol	Entry	Monomer ratio [DMA:HO-LGOL: diol]	HO-LGOL content [%]^a^^a^	Yield [%]^b^^b^	*M*_n_ [Da]^c^^c^	*M*_w_ [Da]^c^^c^	Đ^c^	DP^d^^d^
BDO	4	100:25:50	34	20	3100	5000	1.63	14.1
	5	100:50:50	49	59	700	4400	5.91	3.3
DOD	6	100:25:50	23	53	2400	5100	2.17	8.0
	7	100:50:50	40	50	2600	9300	3.63	8.8

a Determined via ^1^H NMR analysis (Figures S5–S8).

b Determined by gravimetric measurements.

c Determined via gel permeation chromatography analysis.

d Calculated using eq S2 and Table S1.

Similar to ODO-based terpolymers, a higher HO-LGOL incorporation was observed in polymers with an equimolar ratio between HO-LGOL and the aliphatic diol, for both BDO and DOD-based terpolymers (Figure S16b). Interestingly, the incorporation of HO-LGOL is much higher for BDO-based terpolymers compared to that for ODO-based terpolymers in both tested conditions. For DOD-based terpolymers, both ratios (equimolar and excess of diester) gave comparable results to the corresponding ODO terpolymer, with a slight decrease in HO-LGOL incorporation (Figure S16b). The highest HO-LGOL incorporation observed in this work (49%) was achieved using a 100:50:50 (DMA:HO-LGOL:BDO) monomer feed ratio, as seen in entry 5.

As far as yield is concerned, the results for both tested ratios with DOD hardly differ, and both yields are higher than those obtained with ODO. This is most likely due to the longer carbon chain in DOD compared to ODO, as CaLB prefers long-chain substrates.^23−26^ In contrast, the yields of the BDO-based terpolymers vary much more, as those synthesized with an equimolar monomer ratio (entry 5, Table 3) had a yield of 59%, compared to 20% for those synthesized with excess diester (entry 4, Table 3). Yield is dependent not only on the conditions during synthesis but also on the workup procedure. Given that the polymer in Table 3 (entry 5) is highly disperse with a low *M*_n_ (700 Da), one possible explanation is that this polymer is much less soluble in the methanol antisolvent, resulting in the precipitation of even very short polymer chains during the workup procedure. In fact, the measured DP for this sample is 3.3, indicating the presence of oligomers. This behavior was not observed when the BDO-based terpolyester was synthesized using excess diester (entry 4, Table 3), but in this case, the chemical composition of the polymer is different as it has less HO-LGOL incorporated in the chains (only 34%).

Terpolymers synthesized with DOD had higher molecular weights, comparable to the best results achieved with ODO. Both sets of polymers showed pronounced dispersity, with values between 2 and 4. The terpolymerizations with BDO showed comparable results for both ratios in terms of *M*_w_, but not for *M*_n_, where the equimolar ratio (entry 5, Table 3) had a significantly lower *M*_n_. Excess DMA (entry 4, Table 3) gave the highest DP obtained in this work, 14.1, with a surprisingly low dispersity. Interestingly, this polymer incorporated a significant amount of HO-LGOL at 34%, the highest for those polymers synthesized with excess DMA. Overall, the incorporation of BDO resulted in terpolymers with the highest percentage of HO-LGOL in their structures. Previous work has shown that BDO is not an ideal substrate for CaLB, which prefers longer alkyl substrates.^31^ It is possible that CaLB has a similar selectivity for BDO and HO-LGOL, or at least the degree of preference for each substrate is much closer when compared with the other two systems. Furthermore, the molecular weights are also not insignificant, even though the dispersity is quite high, especially for the 50:50 ratio.

### Thermal Characterization

The thermal properties of the synthesized polyesters were analyzed by TGA and DSC measurements. The results for poly(HO-LGOL adipate-*co*-1,4-butylene adipate), poly(HO-LGOL adipate-*co*-1,8-octylene adipate), and poly(HO-LGOL adipate-*co*-1,12-dodecylene adipate) synthesized with different monomer ratios are reported in Table 4 and can be visualized in Figures S10 and S15. Regarding the terpolymer thermal stability, the onset degradation temperatures (*T*_onset 5%_) were found to be dependent mainly on the molecular weights of the polymers, as the samples characterized by a lower *M*_n_, i.e., those based on the 100:50:50 ratio both for BDO and for ODO, exhibited a lower degradation temperature. This result can indeed be attributed to the reactive end groups, the number of which increases as the molecular weight of the polymer decreases, which can promote depolymerization reactions, as reported in other works.^35^ However, the maximum rate of degradation (*T*_max_) was comparable for all the prepared samples, ranging from 400 to 426 °C, a temperature range typical of the thermal degradation of aliphatic polyesters such as poly(butylene adipate), poly(octylene adipate), and poly(dodecylene adipate).^36^ The typical degradation mechanism reported for these kinds of structures is β-elimination, which occurs when aliphatic hydrogens are on a β-position. It is worth underlining that this finding demonstrates that the incorporation of HO-LGOL into the polymer structure, even in different amounts, did not affect the thermal stability.

**Table 4:** Thermal Analysis Data for Poly(HO-LGOL Adipate-*co*-1,4-butylene Adipate), poly(HO-LGOL Adipate-*co*-1,8-octylene Adipate), and Poly(HO-LGOL Adipate-*co*-1,12-dodecylene Adipate) That were Enzymatically Synthesized using Different Monomer Ratios

Aliphatic diol	Entry	Monomer ratio [DMA:HO-LGOL:diol]	*T*_onset 5%_ [°C]^a^^a^	*T*_max_ [°C]^a^^a^	*T*_c_^b^[°C]^c^	Δ*H*_c_ [J/g]	*T*_g_ [°C]^c^	*T*_m_ [°C]^c^	Δ*H*_m_ [J/g]
BDO	4	100:25:50	285	406	-d	-	–41	-d	-
	5	100:50:50	250	400	-d	-	–37	-d	-
ODO	1	100:25:50	343	418	43	83	–33	55	73
	2	100:50:75	328	419	35	80	–43	49/54	84
	3	100:50:50	251	406	33	56	–27	45/50	55
DOD	6	100:25:50	364	414	43	80	–35	55	87
	7	100:50:50	360	426	33	52	–26	45/50	57

a Determined via TGA measurements. *T*_onset 5%_ was extrapolated from TGA curves as the point where the mass loss was equal to 5%, and *T*_max_ was extrapolated from DTG curves as the points where the mass loss rate was at its maximum.

b First crystallization.

c Determined via DSC measurements. *T*_g_ = glass transition temperature, *T*_m_ = melting temperature, Δ*H*_m_ = melting enthalpy, *T*_c_ = crystallization temperature, Δ*H*_c_ = crystallization enthalpy.

d Not observed.

Table 4 also shows the thermal properties of the synthesized polymers in terms of glass transition temperature (*T*_g_), melting (*T*_m_), and crystallization temperature (*T*_c_), as well as melting (Δ*H*_m_) and crystallization enthalpy (Δ*H*_c_). Considering the above results, it is clear that among the synthesized polymers, only the systems based on BDO did not show any crystallization event, exhibiting only a *T*_g_, while those prepared from ODO and DOD crystallized under the applied cooling conditions. As described in the literature, this result can be explained by the fact that as the linear aliphatic moiety introduced by the diol into the repeating unit of the polymer increases, the chains can organize more easily, facilitating the crystallization process.^36−38^ It is important to emphasize that the results presented above demonstrate the possibility of tuning the properties of the terpolymers by appropriately changing the nature of the diol. It was also found that the *T*_g_ values depend mainly on the terpolymer composition and structure, i.e., the diol used. The molecular weight has less of an influence, as it was observed that systems characterized by the same *M*_n_ (see, for example, 100:50:50 ratio for DOD and 100:50:75 for ODO) have different *T*_g_.

In the case of crystallizable systems, i.e., those based on ODO and DOD, it was found that Δ*H*_c_ and Δ*H*_m_ depend on the specific monomer ratio since the above values change considerably when this parameter is modified. Of particular interest is the comparison of the 100:25:50 and 100:50:50 ratios for the ODO and DOD-based samples, where Δ*H*_c_ decreased from approximately 80 J/g to approximately 50 J/g when the HO-LGOL content was increased, i.e., when moving from the 100:25:50 to 100:50:50 ratio. This result clearly demonstrates that the system structuring is hindered by increasing the content of the bulky monomer in the reaction mixture and that it is possible to finely modify the thermal properties by changing both the monomer composition as well as the ratio.

## Conclusions

In this work, several terpolyesters incorporating the renewable cellulose-based monomer HO-LGOL were developed. The optimal formulation in terms of the ratio between the monomers was determined, which made it possible to obtain terpolymers characterized by a high incorporation of HO-LGOL. Despite the low reactivity of CaLB toward HO-LGOL, terpolyesters with up to 49% HO-LGOL content (with respect to the diol) were obtained. It is also noteworthy that the BDO-based terpolymers synthesized in this work were superior compared to those based on the ODO and DOD (considering HO-LGOL incorporation). It should also be emphasized that most terpolymers synthesized in this work have a markedly high dispersity, which could lead to significant variability in the physical behavior and properties of the materials. When the properties of these polymers are compared with those of other biobased aliphatic polyesters, the rigid HO-LGOL component is expected to confer thermal stability and strength. Polymers based on other rigid biobased monomers, such as isosorbide and 2,5-furandicarboxylic acid, have become increasingly popular in recent years. When compared to the structurally similar LGA-based terpolymers, the polymers synthesized in this work are less thermally stable, but this is likely due to their lower molecular weights. The ability to tune the thermal properties by changing the monomer composition presents an interesting avenue for further study and broadens the potential applications of these materials. Here, Cygnet 2.0 has proven to be a good solvent for this type of polymerization, making it a valid substitute for DPE in this context, as well. Overall, lipase-catalyzed copolymerization of HO-LGOL was shown to be a good strategy for producing thermally stable polyesters, even at low molecular weights.

## Data Availability

The data supporting this article have been included as part of the Supporting Information.

## Supporting Information Available

^1^H spectra of all synthesized polymers, and ^13^C and HSQC NMR spectra of selected polymers, graphs of DSC and TGA curves, exact molecular weight data, equations used to calculate DP, and MALDI spectra of selected polymers (PDF).
