# Supplementary material for: Sustainable Synthesis of Terpolyesters Based on a Levoglucosenone-Derived Cyclic Acetal Diol
Source: ACS Sustain Chem Eng. 2025 Mar 4;13(10):4068–77. doi: 10.1021/acssuschemeng.4c10010 (PMC11921019; doi:10.1021/acssuschemeng.4c10010)
Supplement: Supplementary file 1 — sc4c10010_si_001.pdf [file sc4c10010_si_001.pdf]

Supporting Information

On the Sustainable Synthesis of Terpolyesters Based on a Levoglucosenone-derived Cyclic Acetal Diol

Giacomo Lombardo[1,2], Cicely M. Warne[2,3]*, Giacomo Damonte[1], Anamaria Todea[4], Lajos Nagy[5], Georg M. Guebitz[2,3], Florent Allais[6], Sami Fadlallah[6], Alessandro Pellis[1,*]

[1] University of Genova, Department of Chemistry and Industrial Chemistry, via Dodecaneso 31, 16146, Genova (GE), Italy.

[2] BOKU University, Vienna, Department of Agrobiotechnology, IFA-Tulln, Institute of Environmental Biotechnology, Konrad-Lorenz-Strasse 20, 3430 Tulln an der Donau, Austria.

[3] ACIB GmbH, Konrad-Lorenz-Strasse 20, 3430 Tulln an der Donau, Austria.

[4] University Politehnica Timisoara, Faculty of Chemical Engineering, Biotechnology and Environmental Protection, Vasile Parvan 6, 300223, Timisoara, Romania.

[5] University of Debrecen, Department of Applied Chemistry, Egyetem tér 1, H-4032 Debrecen, Hungary.

[6] URD Agro-Biotechnologies Industrielles (ABI), CEBB, AgroParisTech, Pomacle 51110, France.

Correspondence to:

Ms. Cicely Warne, email: cicely.warne@boku.ac.at

Prof. Alessandro Pellis, email: alessandro.pellis@unige.it

Figures

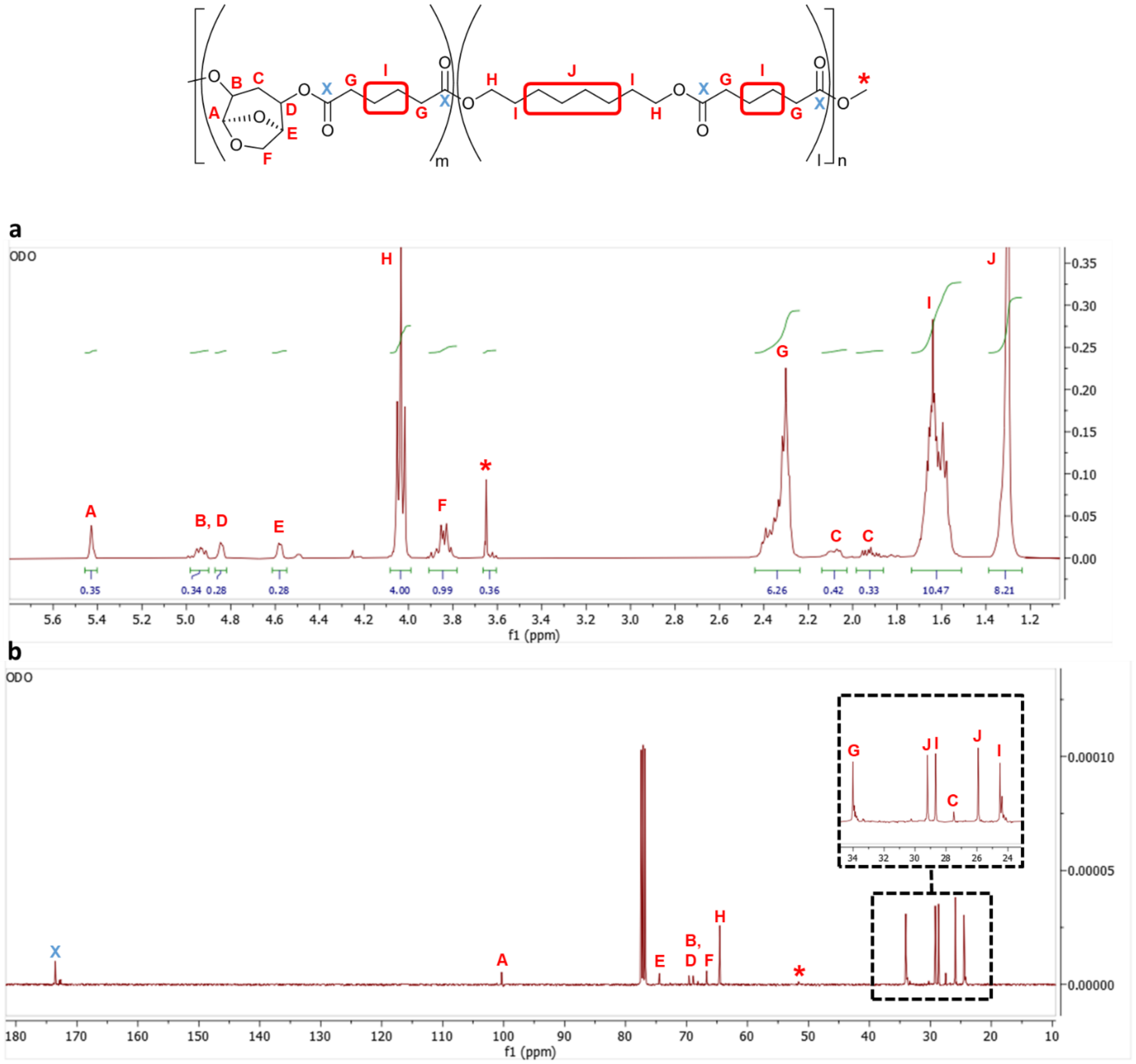

Figure S 1.a. ^{1}H-NMR and b. ^{13}C-NMR spectra of poly(HO-LGOL adipate-co-1,4- octylene adipate) enzymatically synthesized using a 25:50 (HO-LGOL:ODO) monomer ratio using a one-pot procedure, with all peaks assigned.

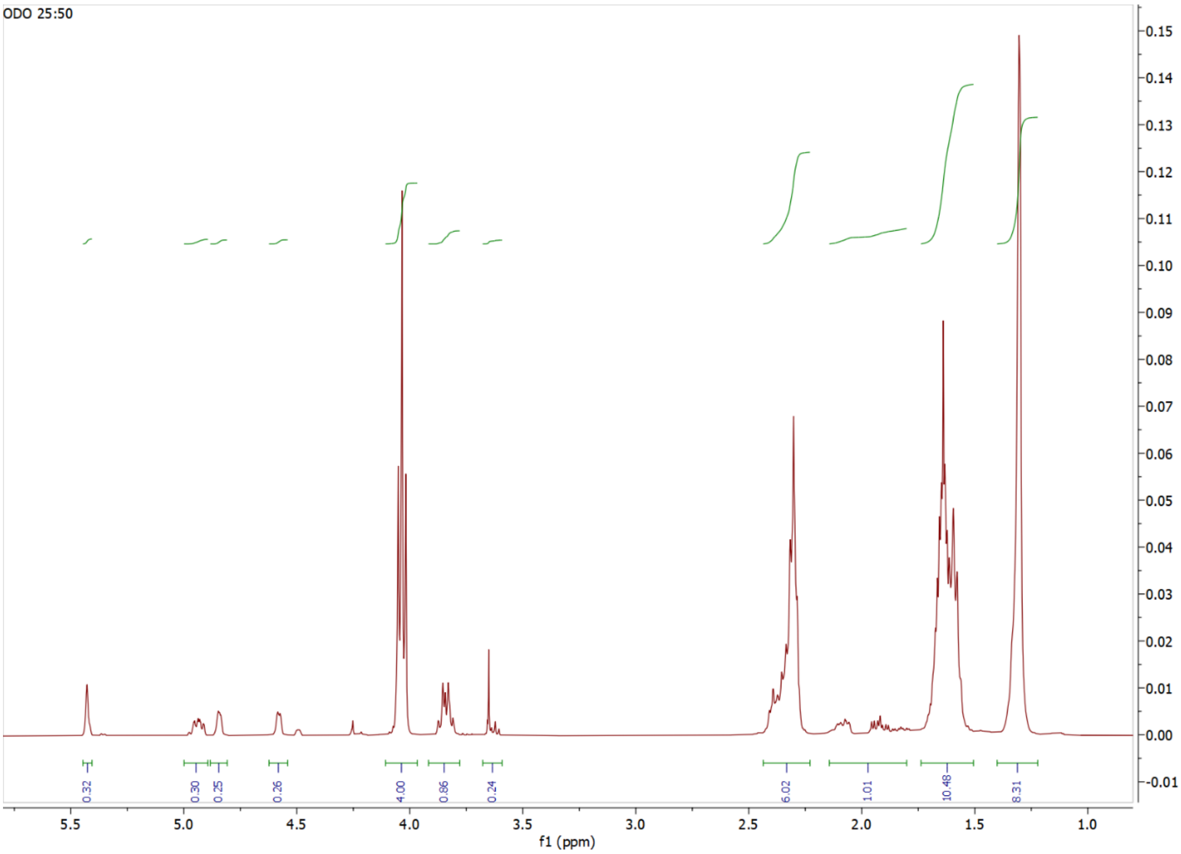

Figure S 2. ¹H-NMR spectra of poly(HO-LGOL adipate-co-1,4- octylene adipate) enzymatically synthesized using 25:50 (HO-LGOL:ODO) monomer ratio.

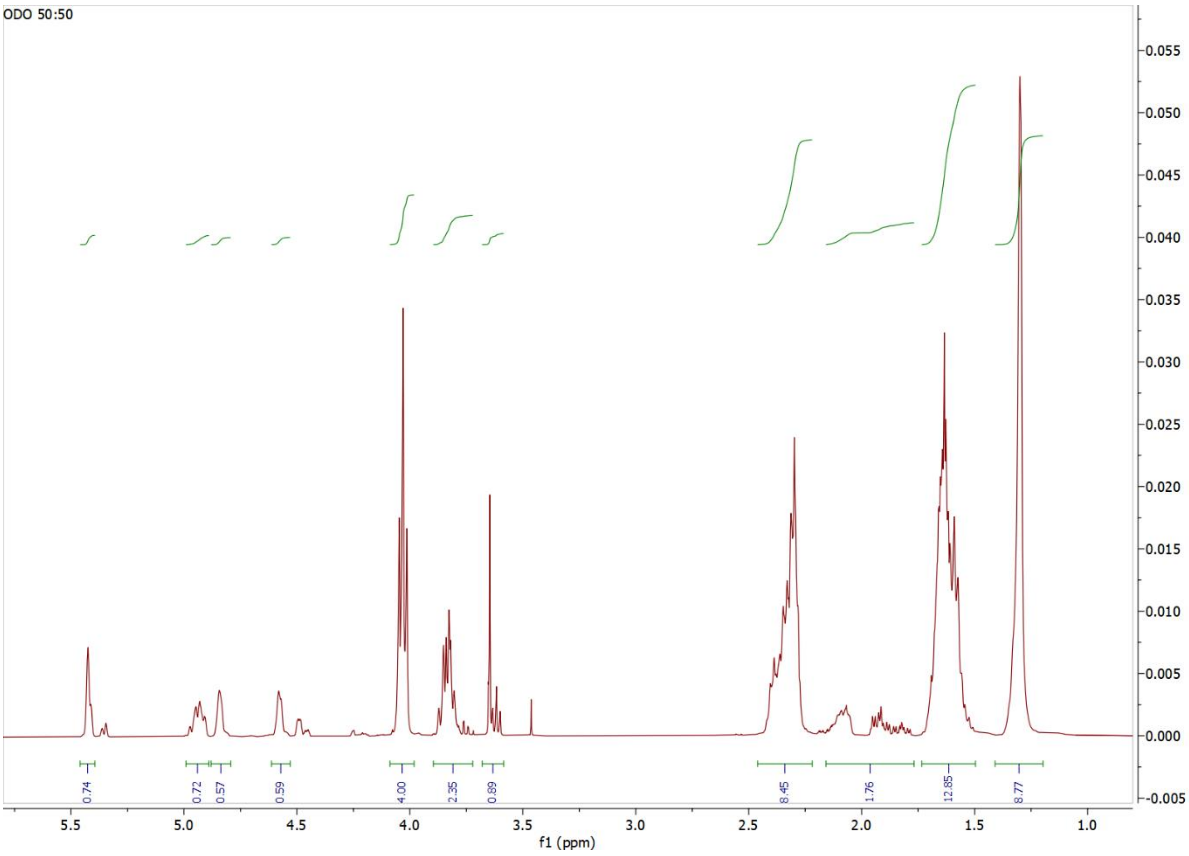

Figure S 3. ¹H-NMR spectra of poly(HO-LGOL adipate-co-1,4- octylene adipate) enzymatically synthesized using a 50:50 (HO-LGOL:ODO) monomer ratio.

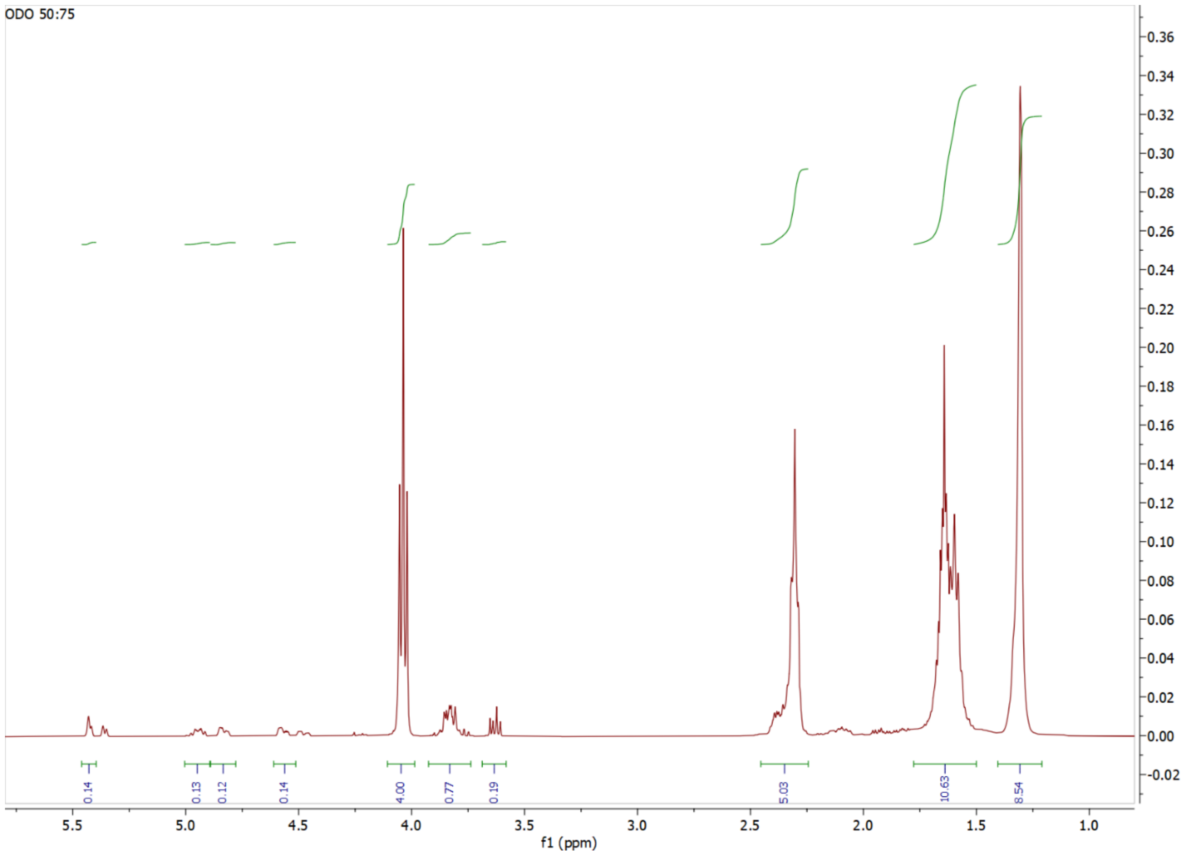

Figure S 4. ^{1}H-NMR spectra of poly(HO-LGOL adipate-co-1,4- octylene adipate) enzymatically synthesized using a 50:75 (HO-LGOL:ODO) monomer ratio.

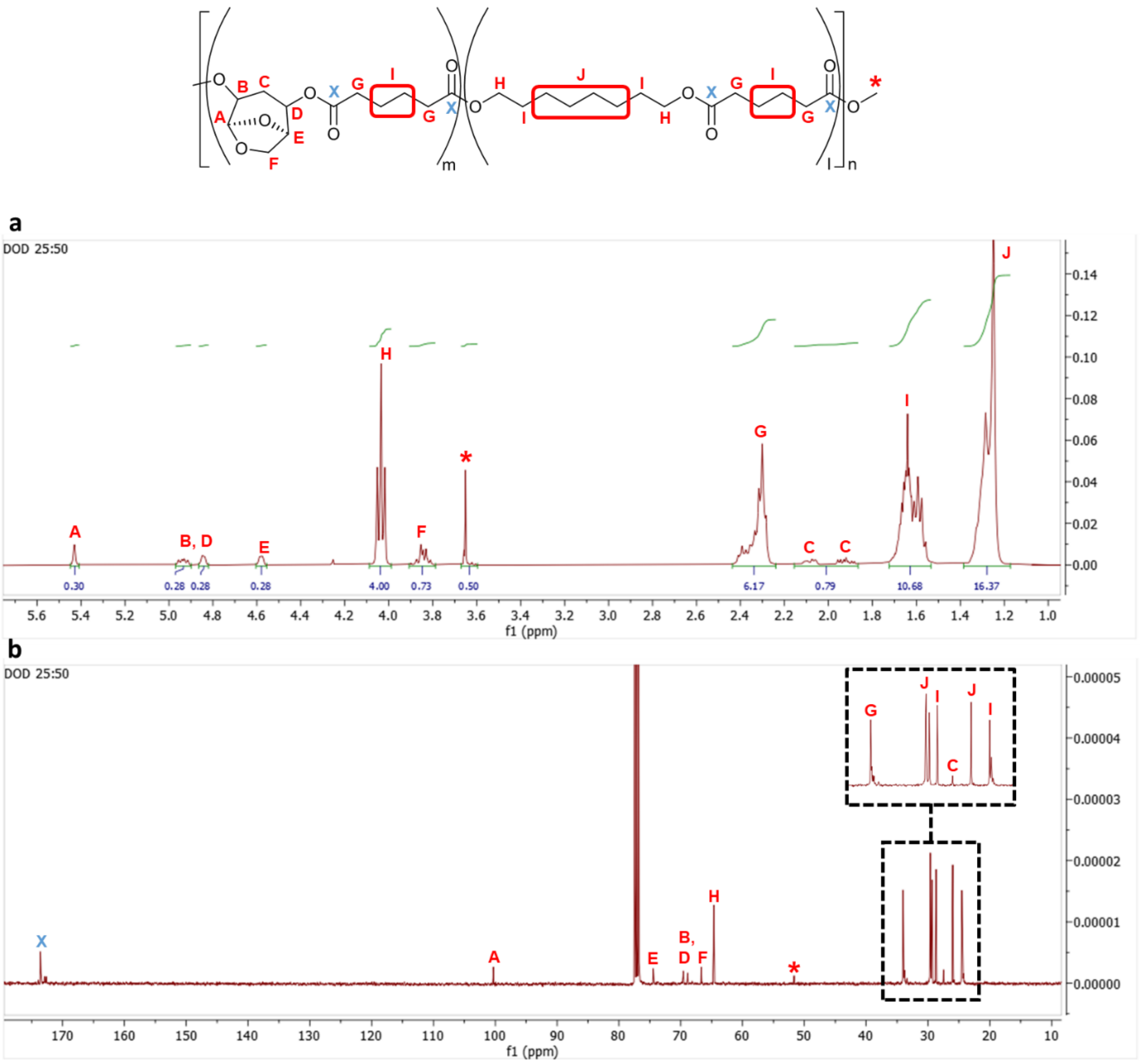

Figure S 5. a. ^{1}H-NMR and b. ^{13}C-NMR spectra of poly(HO-LGOL adipate-co-1,4- dodecylene adipate) enzymatically synthesized using a 25:50 (HO-LGOL:DOD) monomer ratio using a one-pot procedure, with all peaks assigned.

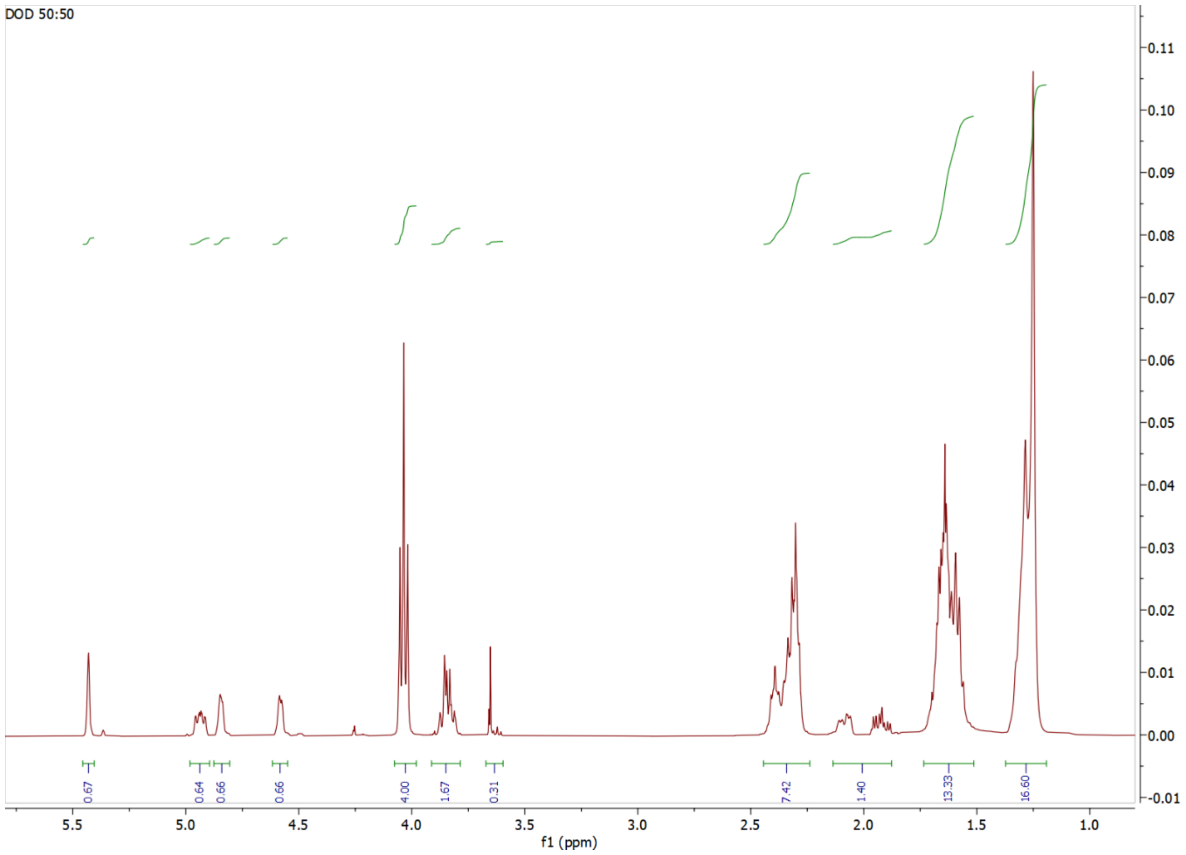

Figure S 6. ¹H-NMR spectra of poly(HO-LGOL adipate-co-1,4- dodecylene adipate) enzymatically synthesized using a 50:50 (HO-LGOL:DOD) monomer ratio.

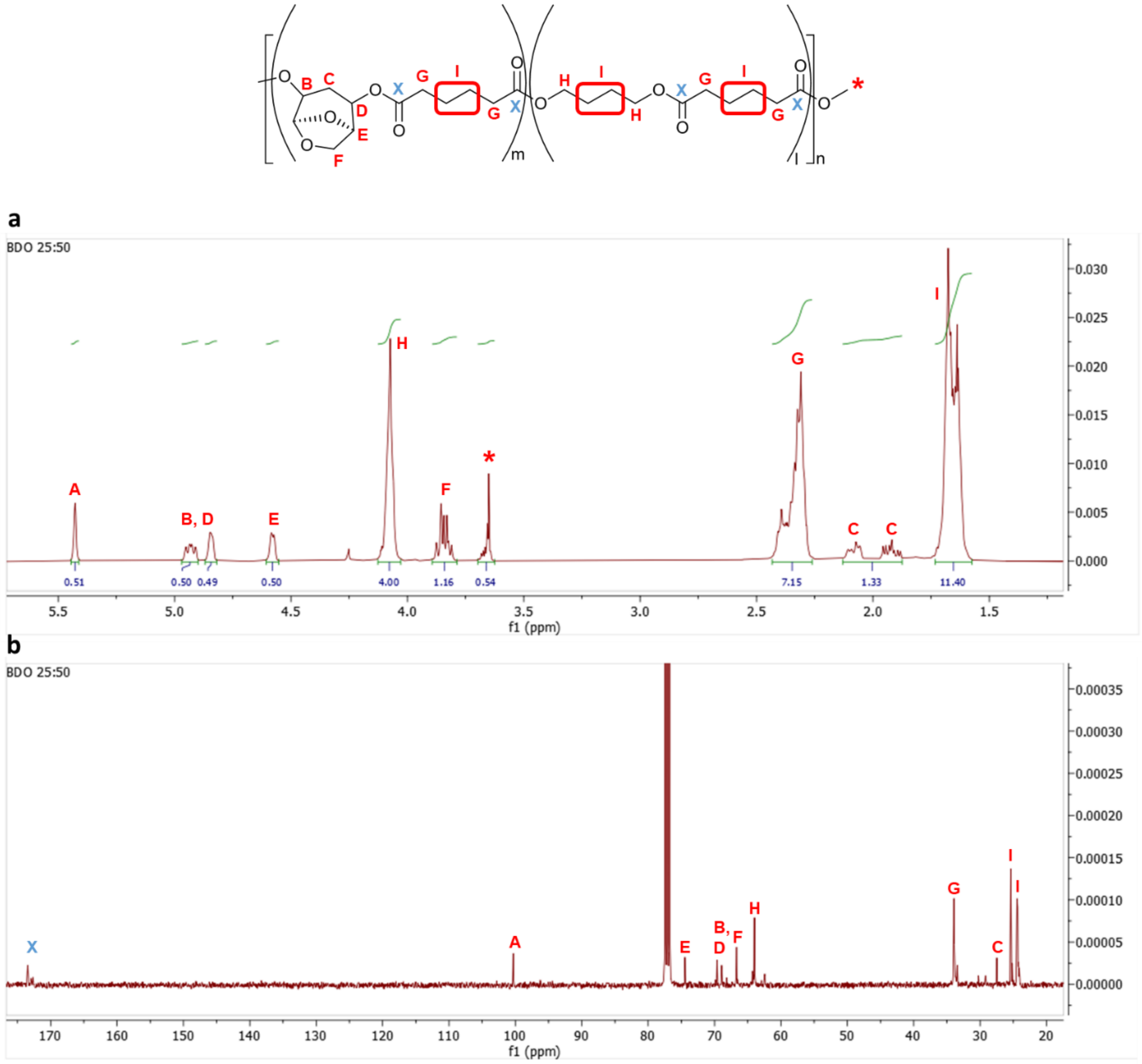

Figure S 7.a. ¹H-NMR and b. ¹³C-NMR spectra of poly(HO-LGOL adipate-co-1,4- butylene adipate) enzymatically synthesized using a 25:50 (HO-LGOL:BDO) monomer ratio, with all peaks assigned.

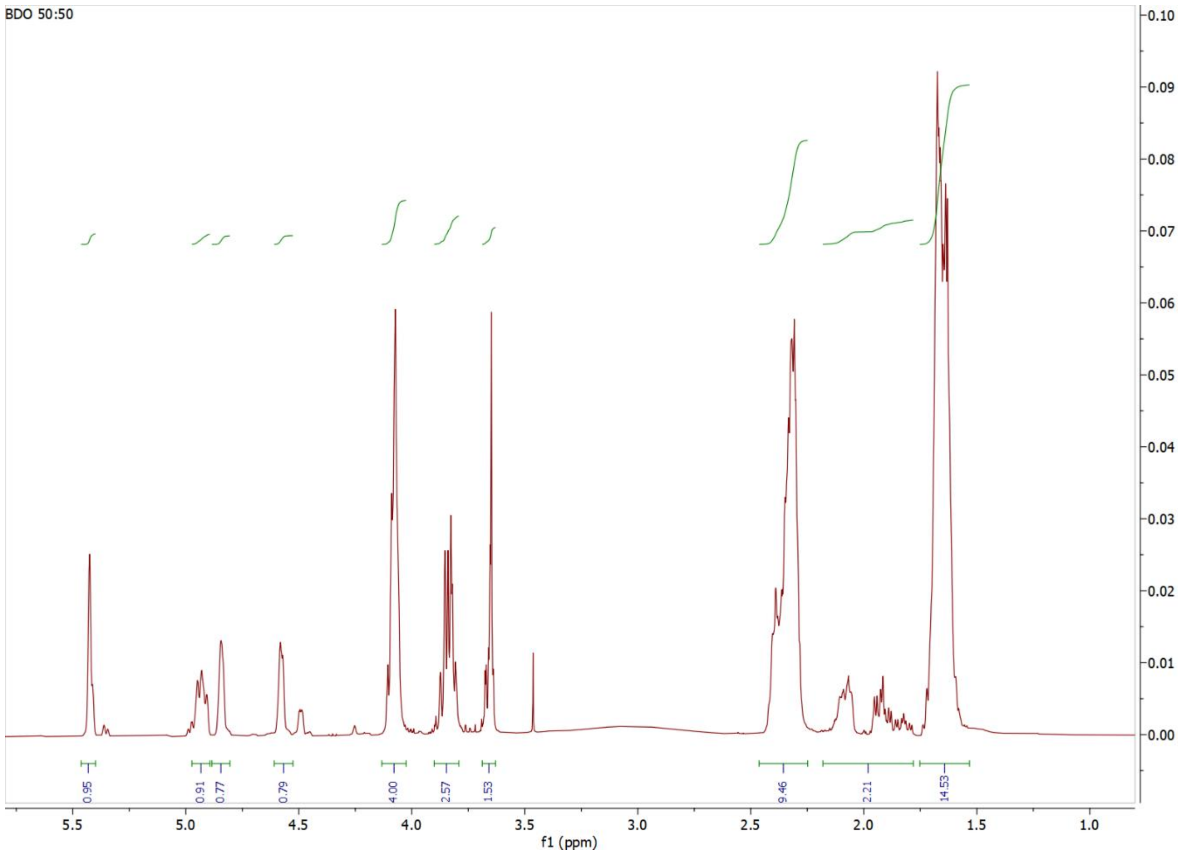

Figure S8. ^{1}H-NMR spectra of poly(HO-LGOL adipate-co-1,4-butylene adipate) enzymatically synthesized using a 50:50 (HO-LGOL:BDO) monomer ratio.

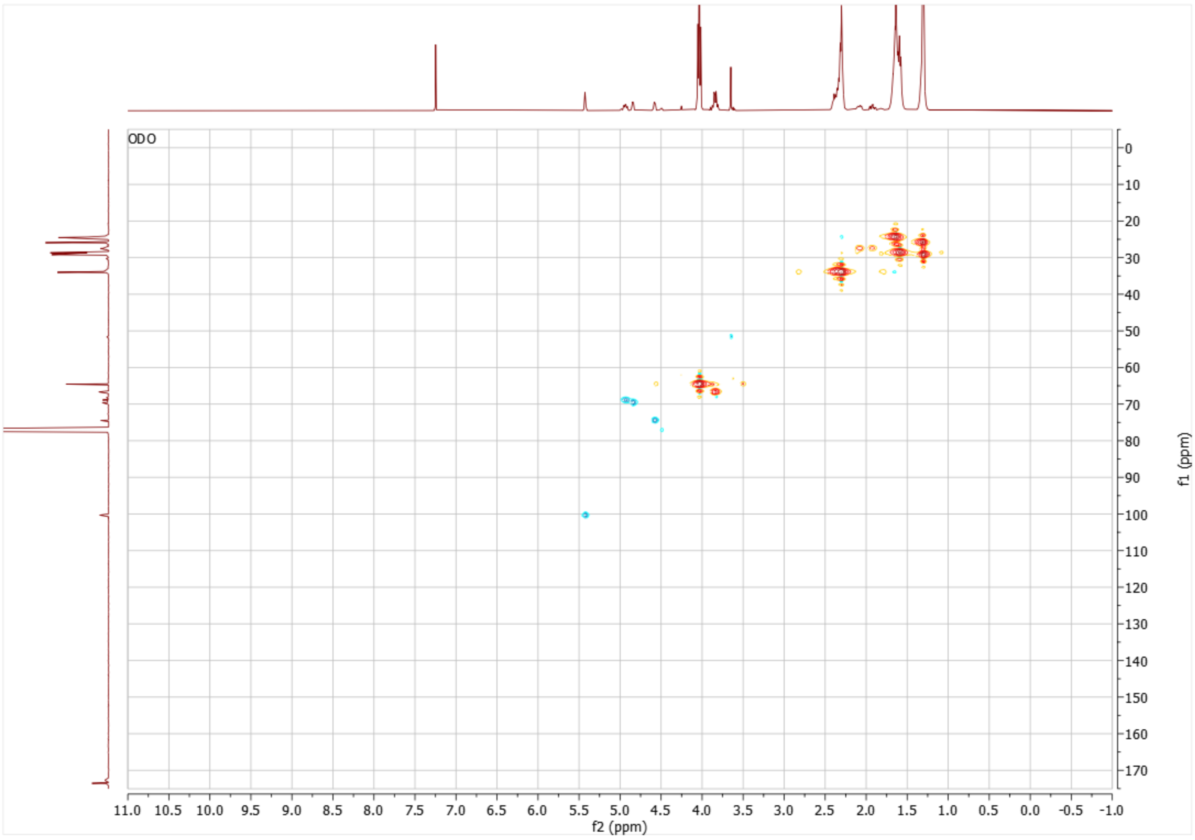

Figure S9. HSQC NMR spectra of poly(HO-LGOL adipate-co-1,4- octylene adipate) enzymatically synthesized using a 25:50 (HO-LGOL:ODO) monomer ratio using a one-pot procedure (Figure S1).

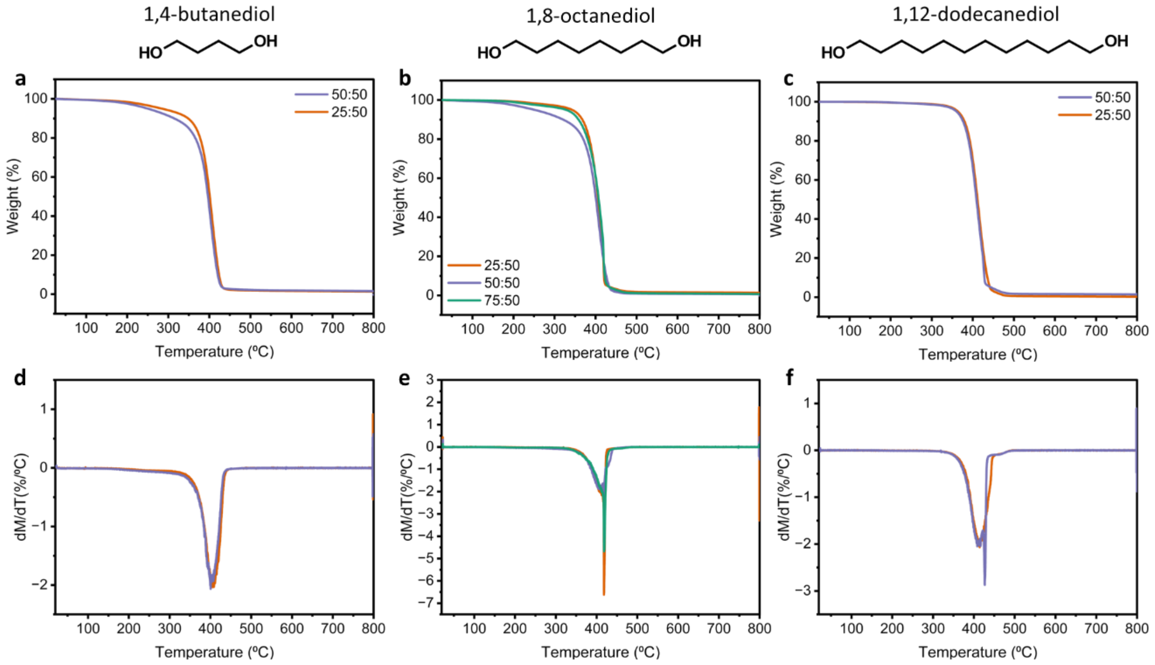

Figure S10. TGA analysis of poly(HO-LGOL adipate-co-1,4-butylene adipate), showing a. weight loss curve and d. its first derivative; poly(HO-LGOL adipate-co-1,8-octylene adipate) showing b. weight loss curve and e. its first derivative, and d. poly(HO-LGOL adipate-co-1,12-dodecylene adipate) showing c. weight loss curve and f. its first derivative.

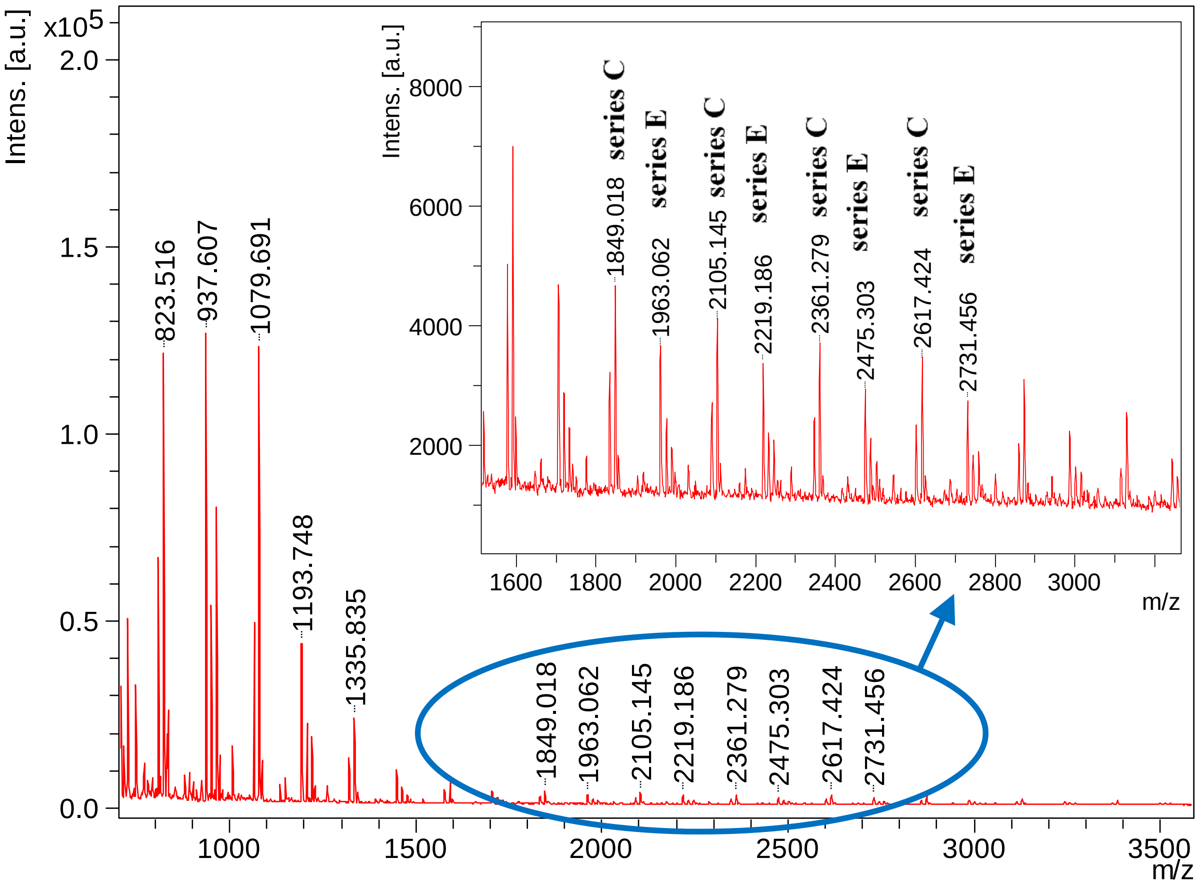

Figure S11. MALDI spectra of poly(HO-LGOL adipate-co-1,8-octylene adipate) enzymatically synthesised using a 100:50:50 [DMA:HO-LGOL:ODO] monomer ratio (entry 3, Table 1) recorded in reflectron mode.

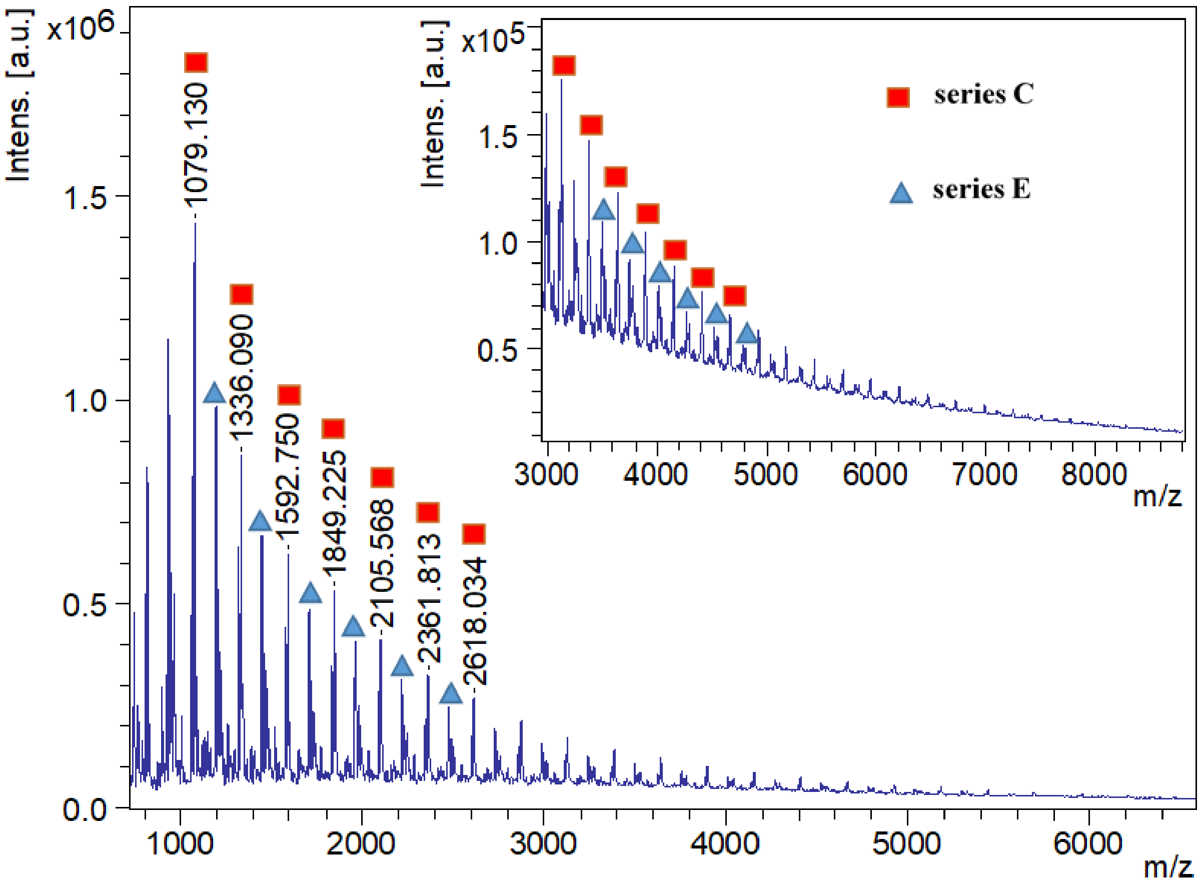

Figure S12. MALDI spectra of poly(HO-LGOL adipate-co-1,8-octylene adipate) enzymatically synthesised using a 100:50:50 [DMA:HO-LGOL:ODO] monomer ratio (entry 3, Table 1) recorded in linear mode. Series C (diol + diester end groups) is indicated by the presence of a red square while series E (carboxylic acid + carboxylic acid end groups) is highlighted with a blue triangle.

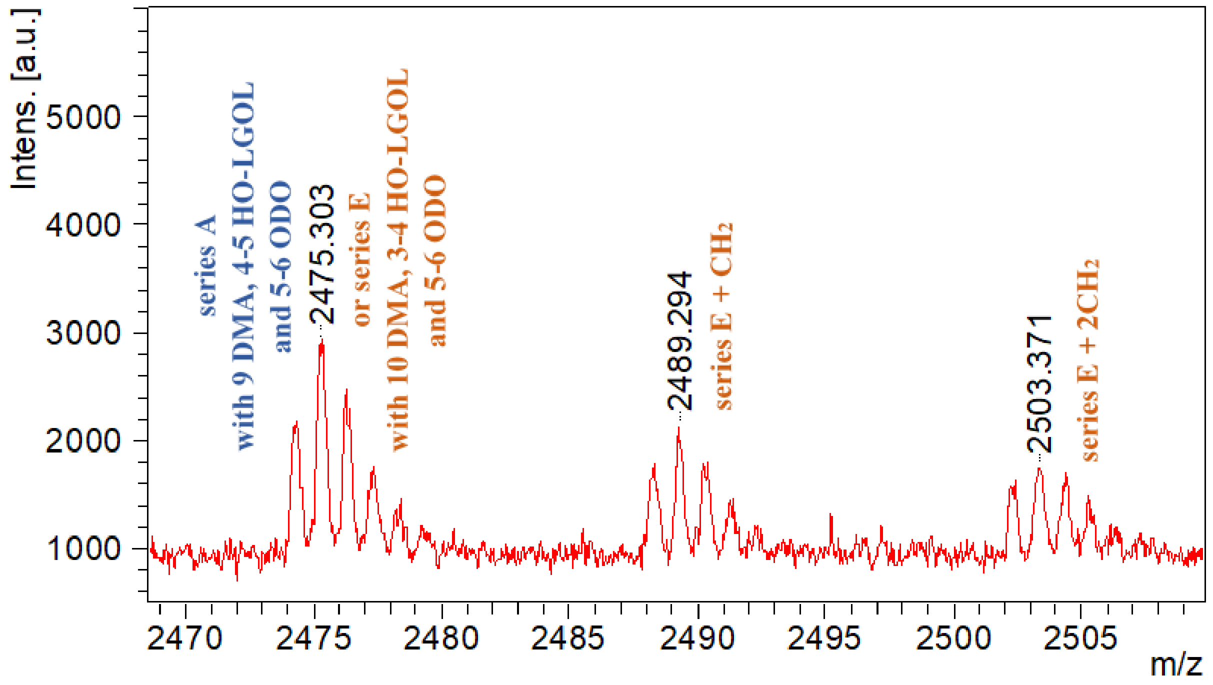

Figure S13. MALDI spectra of poly(HO-LGOL adipate-co-1,8-octylene adipate) enzymatically synthesised using a 100:50:50 [DMA:HO-LGOL:ODO] monomer ratio (entry 3, Table 1) conducted in reflectron mode, showing a close up of m/z 2470 – 2510.

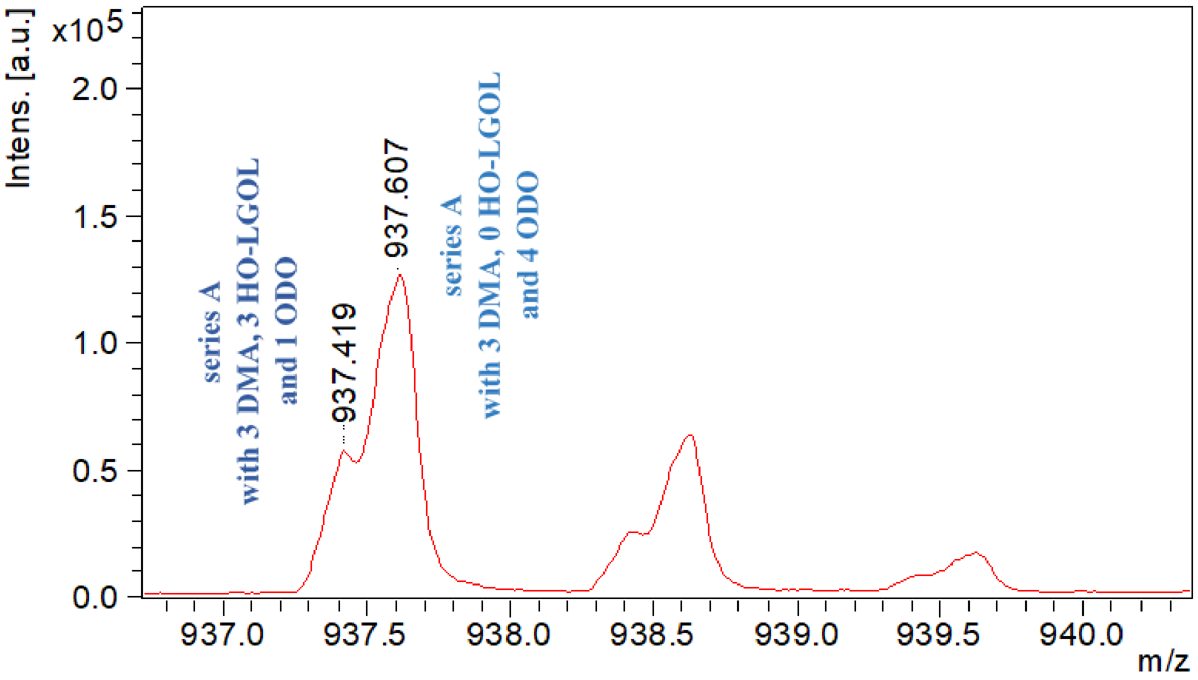

Figure S14. MALDI spectra of poly(HO-LGOL adipate-co-1,8-octylene adipate) enzymatically synthesised using a 100:50:50 [DMA:HO-LGOL:ODO] monomer ratio (entry 3, Table 1) showing a close-up of the peak at m/z 937.

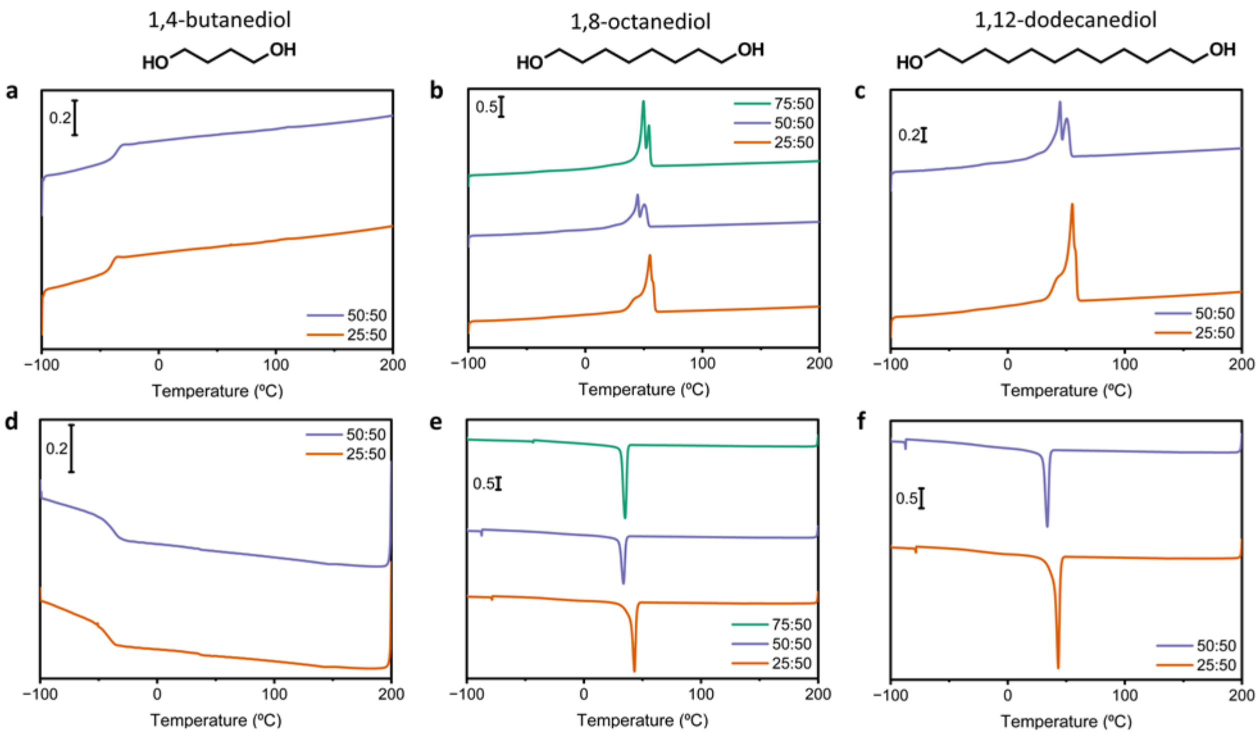

Figure S15. DSC analysis of: poly(HO-LGOL adipate-co-1,4-butylene adipate) showing a. second heating curve and d. first cooling curve; poly(HO-LGOL adipate-co-1,8-octylene adipate) showing d. second heating curve and e. first cooling curve; poly(HO-LGOL adipate-co-1,12-dodecylene adipate) showing c. second heating curve and f. first cooling curve. Scale bars are shown instead of the Y axis, units in W/g.

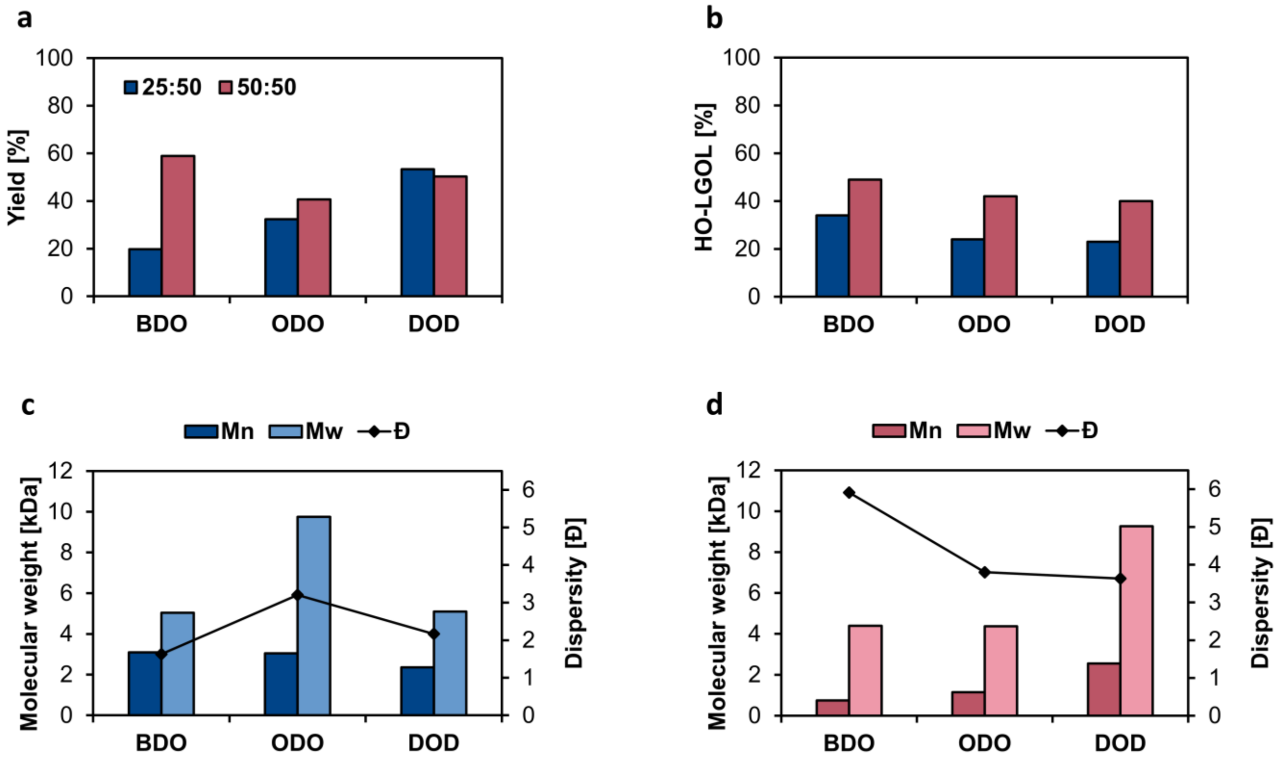

Figure S 16 a. Yields and b. HO-LGOL incorporation (%) of enzymatically synthesized HO-LGOL based terpolymers synthesized with 25:50 and 50:50 (HO-LGOL:aliphatic diol) monomer ratio; and molecular weights of HO-LGOL based terpolymers synthesized using c. 25:50 and d. 50:50 (HO-LGOL:aliphatic diol) ratio.

Tables

Table S1. Exact number average molecular weight data used to calculate DP in Table 1 and 2 using Equations S1 and S2.

**Entry**	**Aliphatic diol**	**Monomer Ratio [DMA:HO-LGOL:ODO]**	**M_n [Da]**
1	ODO	100:25:50	3046
2		100:50:75	2361
3		100:50:50	1151
4	BDO	100:25:50	3095
5		100:50:50	744
6	DOD	100:25:50	2356
7		100:50:50	2551

Equations

$$DP = \frac{M_n}{M_0} \tag{S1}$$

M_0 = Molecular weight of the repeat unit (Da)

$$DP = \frac{M_n}{\left(M_{xy} \times I\right) + \left(M_{zy} \times (1 - I)\right)} \tag{S2}$$

M_{xy} = Molecular weight of the HO-LGOL-DMA repeat unit (Da)
M_{zy} = Molecular weight of the aliphatic diol-DMA repeat unit (Da)
I = HO-LGOL incorporation (%)
